# MAVS Antagonizes Human Stem Cell Senescence as a Mitochondrial Stabilizer

**DOI:** 10.34133/research.0192

**Published:** 2023-07-27

**Authors:** Cui Wang, Kuan Yang, Xiaoqian Liu, Si Wang, Moshi Song, Juan Carlos Izpisua Belmonte, Jing Qu, Guang-Hui Liu, Weiqi Zhang

**Affiliations:** ^1^ CAS Key Laboratory of Genomic and Precision Medicine, Beijing Institute of Genomics, Chinese Academy of Sciences and China National Center for Bioinformation, Beijing 100101, China.; ^2^ University of Chinese Academy of Sciences, Beijing 100049, China.; ^3^ Sino-Danish College, University of Chinese Academy of Sciences, Beijing 101408, China.; ^4^ State Key Laboratory of Stem Cell and Reproductive Biology, Institute of Zoology, Chinese Academy of Sciences, Beijing 100101, China.; ^5^ Institute for Stem Cell and Regeneration, Chinese Academy of Sciences, Beijing 100101, China.; ^6^ Beijing Institute for Stem Cell and Regenerative Medicine, Beijing 100101, China.; ^7^ Advanced Innovation Center for Human Brain Protection, National Clinical Research Center for Geriatric Disorders, Xuanwu Hospital Capital Medical University, Beijing 100053, China.; ^8^ State Key Laboratory of Membrane Biology, Institute of Zoology, Chinese Academy of Sciences, Beijing 100101, China.; ^9^ Altos Labs, Inc., San Diego, CA 94022, USA.

## Abstract

Mitochondrial dysfunction is a hallmark feature of cellular senescence and organ aging. Here, we asked whether the mitochondrial antiviral signaling protein (MAVS), which is essential for driving antiviral response, also regulates human stem cell senescence. To answer this question, we used CRISPR/Cas9-mediated gene editing and directed differentiation techniques to generate various MAVS-knockout human stem cell models. We found that human mesenchymal stem cells (hMSCs) were sensitive to MAVS deficiency, as manifested by accelerated senescence phenotypes. We uncovered that the role of MAVS in maintaining mitochondrial structural integrity and functional homeostasis depends on its interaction with the guanosine triphosphatase optic atrophy type 1 (OPA1). Depletion of MAVS or OPA1 led to the dysfunction of mitochondria and cellular senescence, whereas replenishment of MAVS or OPA1 in MAVS-knockout hMSCs alleviated mitochondrial defects and premature senescence phenotypes. Taken together, our data underscore an uncanonical role of MAVS in safeguarding mitochondrial homeostasis and antagonizing human stem cell senescence.

## Introduction

The mitochondrial antiviral signaling protein termed MAVS, a mitochondrial membrane protein, is essential for the anti-RNA viral response [1–5]. In canonical pathways, MAVS is activated by upstream receptors to form prion-like aggregates and functions as a scaffold to recruit the TANK-binding kinase 1, and inhibitor of κB kinase complex, leading to activation of downstream antiviral pathways [6–13]. Notably, MAVS is a protein almost ubiquitously expressed in mitochondria [2]. However, whether MAVS interacts with other mitochondrial components to affect mitochondrial functions remains poorly investigated.

Mitochondrial dysfunction is a hallmark of aging and is closely interconnected to stem cell exhaustion and cellular senescence [14–25]. In general, aging-associated mitochondrial dysfunction is defined as the impairment of mitochondrial morphology and function, characterized by an increase in fragmented mitochondria due to dysregulation of fission, fusion, and mitophagy, as well as accumulated mitochondrial DNA (mtDNA) mutations and increased mitochondrial mass, accompanied by decreased respiratory capacity, damaged mitochondrial membrane potential, and enhanced levels of reactive oxygen species (ROS) [26–34]. Emerging evidence also suggests that mitochondrial activity is mechanistically linked to stem cell exhaustion, including in a type of adult stem cells called mesenchymal stem cells (MSCs), which are resident in multiple tissues and possess capabilities of both self-renewal and linage differentiation into chondrocytes, osteoblasts, and adipocytes [35–37]. While understanding the role of mitochondria in regulating cellular senescence is important to the aging field, key stabilizers of mitochondria that counteract aging have not been thoroughly investigated. As MAVS is a key component of the molecular machinery at the mitochondrial membrane, we were intrigued by the notion that MAVS, by regulating mitochondrial homeostasis, could eventually control senescence-related outcomes.

In this study, we used the CRISPR/Cas9-mediated gene editing and directed in vitro stem cell differentiation techniques, to generate *MAVS*-specific knockout human embryonic stem cells (hESCs), human MSCs (hMSCs), and human neural stem cells (hNSCs). We found that genetic inactivation of MAVS in hMSCs compromises mitochondrial structure and integrity in a way that accelerates senescence. Mechanistically, the absence of MAVS destabilized mitochondrial optic atrophy type 1 (OPA1), a dynamin-like guanosine triphosphatase localized in the mitochondrial membrane required for fusion, leading to mitochondrial dysfunction and, thereby, hMSC senescence. Altogether, our study identifies an uncanonical role of MAVS in maintaining mitochondrial homeostasis and antagonizing cellular senescence in hMSCs.

## Results

### MAVS is dispensable for hESC homeostasis

**Fig. 1.**
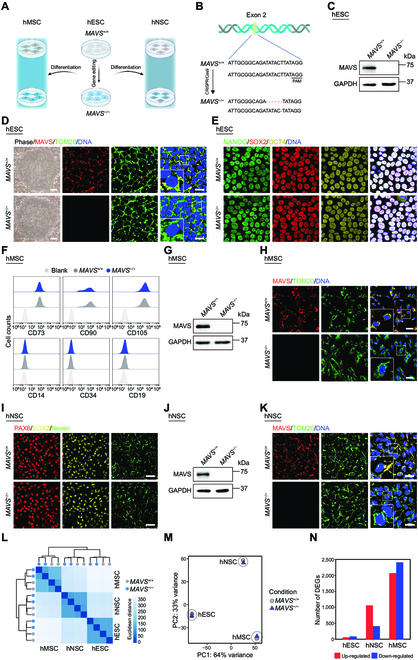
Generation and characterization of *MAVS*-deficient pluripotent stem cells. (A) Schematic workflow showing the generation of *MAVS*^+/+^ and *MAVS*^−/−^ hMSCs and hNSCs from hESCs. (B) Schematic illustration of *MAVS* gene editing in exon 2 using CRISPR/Cas9-mediated nonhomologous end joining in hESCs. (C) Western blotting analysis of MAVS protein in *MAVS*^+/+^ and *MAVS*^−/−^ hESCs. GAPDH was used as a loading control. (D) Left: Phase-contrast images of *MAVS*^+/+^ and *MAVS*^−/−^ hESCs. Scale bar, 250 μm. Right: Immunofluorescence staining of MAVS and TOM20 in *MAVS*^+/+^ and *MAVS*^−/−^ hESCs. Scale bar, 25 μm. Insets show the zoomed-in images. (E) Immunofluorescence staining of pluripotency markers NANOG, SOX2, and OCT4 in *MAVS*^+/+^ and *MAVS*^−/−^ hESCs. Scale bar, 25 μm. (F) FACS analysis of canonical markers of hMSCs, including CD73, CD90, and CD105, as well as irrelevant markers CD14, CD19, and CD34 in *MAVS*^+/+^ and *MAVS*^−/−^ hMSCs. Cells unstained were used as the blank control. (G) Western blotting analysis of MAVS protein in *MAVS*^+/+^ and *MAVS*^−/−^ hMSCs. GAPDH was used as a loading control. (H) Immunofluorescence staining of MAVS and TOM20 in *MAVS*^+/+^ and *MAVS*^−/−^ hMSCs. Scale bar, 50 μm. Insets show the zoomed-in images. (I) Immunofluorescence staining of hNSC markers PAX6, SOX2, and Nestin in *MAVS*^+/+^ and *MAVS*^−/−^ hNSCs. Scale bar, 25 μm. (J) Western blotting analysis of MAVS protein in *MAVS*^+/+^ and *MAVS*^−/−^ hNSCs. GAPDH was used as a loading control. (K) Immunofluorescence staining of MAVS and TOM20 in *MAVS*^+/+^ and *MAVS*^−/−^ hNSCs. Scale bar, 25 μm. Insets show the zoomed-in images. (L) Heatmap showing the Euclidean distance between replicates of RNA-seq in *MAVS*^+/+^ and *MAVS*^−/−^hESCs, hNSCs, and hMSCs. The color key of the Euclidean distance from blue to white indicates strong to weak correlation. (M) Principal component (PC) analysis showing the reproducibility of RNA-seq between replicates in *MAVS*^+/+^ and *MAVS*^−/−^ hESCs, hNSCs, and hMSCs. (N) Bar plot showing the number of up-regulated (red) and down-regulated (blue) DEGs between *MAVS*^+/+^ and *MAVS*^−/−^ hESCs, hNSCs, and hMSCs.

To evaluate the roles of MAVS in regulating stem cell homeostasis and cellular senescence, we generated *MAVS-*specific knockout hESCs (referred to as *MAVS*^−/−^ hESCs) utilizing CRISPR/Cas9-mediated gene editing (Fig. 1A). Specifically, we designed small guide RNA (sgRNA) to target the second exon of the *MAVS* gene and verified by sequencing that a double heterozygous deletion in the *MAVS* gene was present in positive hESC clones, resulting in a frameshift mutation and premature stop codon, and absence of MAVS protein (Fig. 1B). To rule out off-target effects of the sgRNA, the predicted genomic loci with high off-target potential were amplified via polymerase chain reaction (PCR) followed by sequencing, and there was no off-target cleavage detected at these loci in *MAVS*^−/−^ hESCs (Fig. S1A). We then used western blotting to verify the successful loss of MAVS full-length protein (Fig. 1C). Immunofluorescence staining showed that MAVS colocalized with TOM20, a mitochondrial outer membrane protein marker, in *MAVS*^+/+^ hESCs, but no MAVS signal presented in *MAVS*^−/−^ hESCs (Fig. 1D). Thus far, we have successfully generated *MAVS-*specific knockout hESCs.

Upon investigating whether MAVS deficiency affects general hESC features, we verified that the cell morphology of *MAVS*^−/−^ hESCs was the same as that of *MAVS*^+/+^ hESCs (Fig. 1D). In addition, NANOG, SOX2, and OCT4, the pluripotency markers, were well maintained in *MAVS*^−/−^ hESCs (Fig. 1E). Consistently, we detected the presence of endoderm, mesoderm, and ectoderm in the teratoma assay, validating in vivo differentiation ability of *MAVS*^−/−^ hESCs (Fig. S1B). Finally, we found that *MAVS*^−/−^ hESCs maintained a normal karyotype (Fig. S1C). These data indicate that MAVS is dispensable for maintaining regular hESC properties.

### Generation of MAVS-deficient hMSCs and hNSCs

To investigate whether MAVS deficiency affects the homeostasis of human adult stem cells, we performed directed differentiation of *MAVS*^+/+^ and *MAVS*^−/−^ hESCs into hMSCs and hNSCs, respectively (Fig. 1A). We purified *MAVS*^+/+^ and *MAVS*^−/−^ hMSCs via fluorescence-activated cell sorting (FACS) using CD105, CD73, and CD90, the hMSC surface markers that are specifically expressed in hMSCs, rather than the irrelevant markers, such as CD14, CD19, and CD34 (Fig. 1F). The MAVS protein was absent in *MAVS*^−/−^ hMSCs as verified by western blotting (Fig. 1G). In addition, by costaining with the mitochondrial marker TOM20, we found that MAVS colocalized with TOM20 at mitochondria in *MAVS*^+/+^ hMSCs, but the MAVS signal was absent in *MAVS*^−/−^ hMSCs (Fig. 1H). Similarly, all 3 hNSC markers, including PAX6, SOX2, and Nestin, were expressed at similar levels across *MAVS*^−/−^ hNSCs and *MAVS*^+/+^ hNSCs (Fig. 1I), whereas MAVS deficiency was confirmed by western blotting and immunofluorescence staining in *MAVS*^−/−^ hNSCs (Fig. 1J and K).

To roughly compare the roles of MAVS in regulating the homeostasis of various stem cells, we performed transcriptomic sequencing (RNA sequencing [RNA-seq]) in *MAVS*^+/+^ and *MAVS*^−/−^ hESCs, hNSCs, and hMSCs (Fig. 1L and M). We found that the number of differentially expressed genes (DEGs) between *MAVS*^+/+^ and *MAVS*^−/−^ hMSCs was markedly higher than that of hESCs and hNSCs counterparts (Fig. 1N). These results indicate that MAVS likely exerts a cell-type-specific role in maintaining stem cell function and that hMSCs appear to be more vulnerable to the absence of MAVS.

### Depletion of MAVS accelerates cellular senescence of hMSCs

**Fig. 2.**
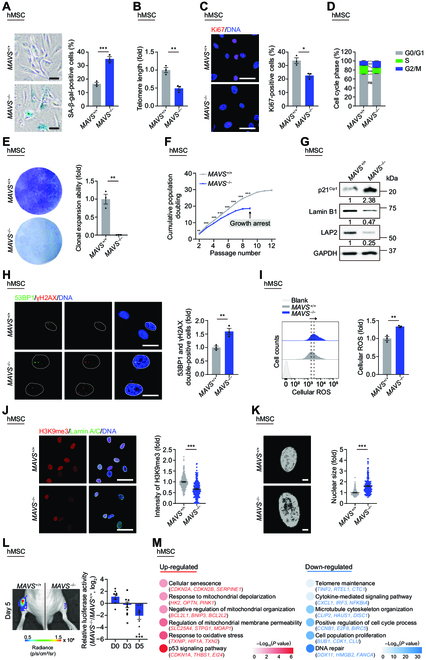
Depletion of MAVS accelerates cellular senescence of hMSCs. (A) SA-β-gal staining of *MAVS*^+/+^ and *MAVS*^−/−^ hMSCs at LP (P8). Scale bar, 50 μm. Data are presented as the means ± SEM. *n* = 3 biological replicates. ***, *P* < 0.001 (*t* test). (B) Telomere length analysis of *MAVS*^+/+^ and *MAVS*^−/−^ hMSCs at LP (P8) by qPCR. Data are presented as the means ± SEM. *n* = 3 technological replicates. **, *P* < 0.01 (*t* test). (C) Immunofluorescence staining of Ki67 in *MAVS*^+/+^ and *MAVS*^−/−^ hMSCs at LP (P8). Scale bar, 50 μm. Data are presented as the means ± SEM. *n* = 3 biological replicates. *, *P* < 0.05 (*t* test). (D) Bar plot of cell cycle analysis in *MAVS*^+/+^ and *MAVS*^−/−^ hMSCs at LP (P8). Data are presented as the means ± SEM. *n* = 3 biological replicates. ns, not significant; **, *P* < 0.01; ***, *P* < 0.001 (*t* test). (E) Clonal expansion analysis of *MAVS*^+/+^ and *MAVS*^−/−^ hMSCs at LP (P8). Data are presented as the means ± SEM. *n* = 3 biological replicates. **, *P* < 0.01 (*t* test). (F) Growth curves of *MAVS*^+/+^ and *MAVS*^−/−^ hMSCs. Data are presented as the means ± SEM. *n* = 3 biological replicates. **, *P* < 0.01; ***, *P* < 0.001 (*t* test). (G) Western blotting analysis of aging-related markers p21^Cip1^, Lamin B1, and LAP2 in *MAVS*^+/+^ and *MAVS*^−/−^ hMSCs at LP (P8). GAPDH was used as a loading control. The numbers below represent relative protein levels. The statistical analysis is shown in Fig. S2E. (H) Immunofluorescence staining of 53BP1 and γH2AX in *MAVS*^+/+^ and *MAVS*^−/−^ hMSCs at LP (P8). Scale bar, 25 μm. Data are presented as the means ± SEM. *n* = 3 biological replicates. **, *P* < 0.01 (*t* test). (I) FACS analysis of total cellular ROS levels by staining with the free radical sensor H2DCFDA in *MAVS*^+/+^ and *MAVS*^−/−^ hMSCs at middle passage (MP, P6). Dashed lines indicate the position of mean fluorescent intensity (MFI). Cells unstained were used as the blank control. Data are presented as the means ± SEM. *n* = 3 biological replicates. **, *P* < 0.01 (*t* test). (J) Left: Immunofluorescence staining of H3K9me3 and Lamin A/C in *MAVS*^+/+^ and *MAVS*^−/−^ hMSCs at LP (P8). Scale bar, 50 μm. Right: Statistical analysis of mean fluorescence intensity of H3K9me3. *n* = 200 cells. ***, *P* < 0.001 (*t* test). (K) Left: Nuclear DNA staining in *MAVS*^+/+^ and *MAVS*^−/−^ hMSCs at LP (P8). Scale bar, 10 μm. Right: Statistical analysis of nuclear size. *n* = 180 cells. ***, *P* < 0.001 (*t* test). (L) Analysis of luciferase activities in TA muscles of immunodeficient mice transplanted with *MAVS*^+/+^ (left) or *MAVS*^−/−^ hMSCs (right) at MP (P6) at Day 0, 3, and 5 after implantation. Data calculated by the ratios of log_2_(*MAVS*^−/−^/*MAVS*^+/+^) are presented as the means ± SEM. *n* = 8 mice. *, *P* < 0.05; ***, *P* < 0.001 (*t* test). (M) Point plot showing GO terms and pathways enriched by up-regulated (red) and down-regulated (blue) DEGs between *MAVS*^+/+^ and *MAVS*^−/−^ hMSCs at LP (P8). The color keys from gray to red or blue indicate low to high enrichment levels.

Upon further inspection of the cellular characteristics of *MAVS*^+/+^ and *MAVS*^−/−^ hMSCs, we detected a series of senescence-associated defects in *MAVS*^−/−^ hMSCs, including an increased percentage of senescence-associated β-galactosidase (SA-β-gal)-positive cells (Fig. 2A), a widely used biomarker for senescent cells [38,39]. *MAVS*^−/−^ hMSCs also exhibited shortened telomere length, another important feature of senescent cells (Fig. 2B). Although we had not detected any noticeable proliferation differences across *MAVS*^+/+^ and *MAVS*^−/−^ hESCs and hNSCs, manifested as comparable percentages of Ki67-positive cells and cell cycle distribution via FACS analysis (Fig. S2A to C), we observed a marked decrease of the percentage of Ki67-positive cells and reduction of the proportion of S-phase cells in *MAVS*^−/−^ relative to *MAVS*^+/+^ hMSCs (Fig. 2C and D), indicating that MAVS depletion compromises cell proliferation in hMSCs. Consistent with these data, we also observed compromised clonal expansion ability and premature growth arrest upon serial passaging in *MAVS*^−/−^ hMSCs (Fig. 2E and F).

Additionally, we observed that the protein level of the senescence marker p21^Cip1^ and the mRNA level of p16^INK4A^ (*CDKN2A*) increased in *MAVS*^−/−^ hMSCs (Fig. 2G and Fig. S2D and E). As it is known that increased levels of ROS [40] and DNA damage [41] are also characteristics of cellular senescence, we investigated levels of ROS and DNA damage upon MAVS depletion. Immunofluorescence staining of DNA damage markers p53-binding protein 1 (53BP1) and γH2AX showed increased DNA damage levels in *MAVS*^−/−^ hMSCs (Fig. 2H). We also observed enhanced total cellular ROS levels in *MAVS*^−/−^ hMSCs via FACS analysis (Fig. 2I). Corresponding to the fact that senescent cells exhibit abnormal nuclear morphology with loss of heterochromatin stability and nuclear lamina integrity [42–46], we also observed reduced protein and transcriptional levels of Lamin B1 (*LMNB1*) and lamina-associated protein LAP2 (*TMPO*) (Fig. 2G and Fig. S2D to F), and lesser heterochromatin marker H3K9me3 (Fig. 2J), as well as increased nuclear size in *MAVS*^−/−^ hMSCs (Fig. 2K). Moreover, when transplanted into the tibialis anterior (TA) muscles of immunodeficient mice, *MAVS*^−/−^ hMSCs underwent accelerated decay in vivo relative to *MAVS*^+/+^ hMSCs, in line with the accelerated senescence phenotypes in vitro (Fig. 2L). However, *MAVS* deficiency did not compromise the basic cell identity of *MAVS*^−/−^ hMSCs; *MAVS*^−/−^ hMSCs still maintained capacity for chondrogenesis, adipogenesis, and osteogenesis, although slightly decreased differentiation efficacy into chondrocytes and adipocytes was observed in *MAVS*^−/−^ hMSCs (Fig. S2G). In addition, genomic integrity was maintained in *MAVS*^−/−^ hMSCs based on copy number variation (CNV) analysis (Fig. S2H).

Consistent with the accelerated cellular senescence phenotypes (Fig. 2A to L and Fig. S2D to F), transcriptomic analysis demonstrated similar deficiencies in *MAVS*^−/−^ hMSCs (Fig. 2M and Fig. S2I to K). Specifically, Gene Ontology (GO) term analysis demonstrated that genes involved in telomere maintenance (e.g., *TINF2* and *RTEL1*) were down-regulated in *MAVS*^−/−^ hMSCs, in line with the phenotypic observations (Fig. 2M). Moreover, MAVS depletion led to disturbance in pathways implicated in cellular senescence, such as up-regulation of p53 signaling pathway (e.g., *CDKN1A* and *EI24*) and cellular senescence per se (e.g., *CDKN2A*, *CDKN2B*, and *SERPINE1*), as well as down-regulation of DNA repair (e.g., *DDX11* and *FANCA*) and cell cycle (e.g., *CCNB1*, *E2F8*, and *BIRC*5). Collectively, these data suggest that endogenous MAVS has the activity of antagonizing hMSC aging and that knockout of MAVS in hMSCs triggers multifaceted features of senescence.

### MAVS deficiency compromises mitochondrial dynamics

**Fig. 3.**
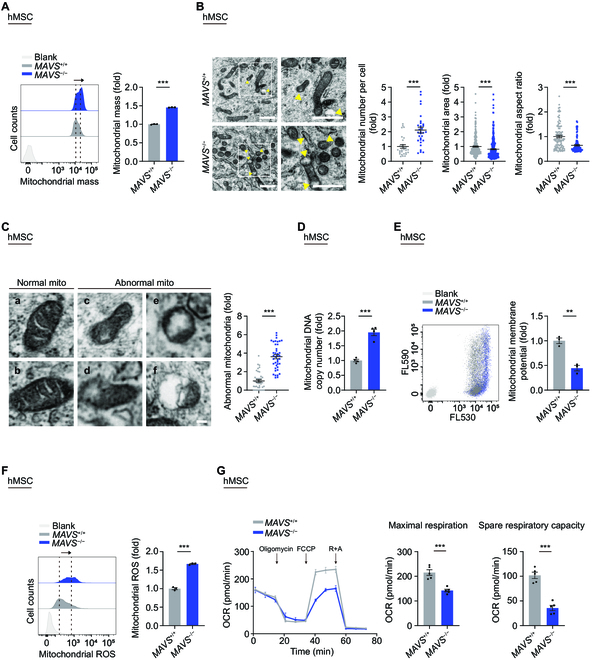
MAVS deficiency compromises mitochondrial dynamics. (A) FACS analysis of mitochondrial mass levels in *MAVS*^+/+^ and *MAVS*^−/−^ hMSCs at LP (P8). Dashed lines indicate the position of MFI. Cells unstained were used as the blank control. Data are presented as the means ± SEM. *n* = 3 biological replicates. ***, *P* < 0.001 (*t* test). (B) Left: TEM analyses of mitochondrial number, mitochondrial area and aspect ratio in *MAVS*^+/+^ and *MAVS*^−/−^ hMSCs at MP (P6). Yellow arrows indicate abnormal mitochondria. Scale bar, 1 μm. Right: Statistical analyses of mitochondrial number per intact cell, mitochondrial area, and aspect ratio. Data are presented as the means ± SEM. *n* = 30 cells; *n* = 300 mitochondria; *n* = 100 mitochondria. ***, *P* < 0.001 (*t* test). (C) Left: Micrographs of normal (a and b) and abnormal (c to f) mitochondria (“mito” indicates mitochondria). The former had typical morphology, and the latter showed severe disorganization of the membrane, including extensive loss of cristae, decreased electron density of the matrix, and dissolution and “bleb” formation. Scale bar, 100 nm. Right: Statistical analysis of relative percentage of abnormal mitochondria. Data are presented as the means ± SEM. *n* = 40 images to determine the relative percentage of abnormal mitochondria in each sample. ***, *P* < 0.001 (*t* test). (D) qPCR analysis of mtDNA copy number in *MAVS*^+/+^ and *MAVS*^−/−^ hMSCs at LP (P8). Data are presented as the means ± SEM. *n* = 4 technological replicates. ***, *P* < 0.001 (*t* test). (E) FACS analysis of mitochondrial membrane potential in *MAVS*^+/+^ and *MAVS*^−/−^ hMSCs at LP (P8) using a fluorescence probe JC-10. Cells unstained were used as the blank control. Data are presented as the means ± SEM. *n* = 3 biological replicates. **, *P* < 0.01 (*t* test). (F) FACS analysis of mitochondrial ROS levels by MitoSOX red staining in *MAVS*^+/+^ and *MAVS*^−/−^ hMSCs at LP (P8). Dashed lines indicate the position of MFI. Cells unstained were used as the blank control. Data are presented as the means ± SEM. *n* = 3 biological replicates. ***, *P* < 0.001 (*t* test). (G) Detection of the OCR in *MAVS*^+/+^ and *MAVS*^−/−^ hMSCs at MP (P6) in response to indicated mitochondrial modulators by Seahorse analysis. Maximal respiration and spare respiratory capacity were calculated by the OCR values. Data are presented as the means ± SEM. *n* = 5 biological replicates. ***, *P* < 0.001 (*t* test).

It is well known that MAVS plays an essential role in driving antiviral innate immunity and stimulating the expression of type I interferons and proinflammatory cytokines in response to RNA virus infection [5,6,47,48]. As expected, the downstream proinflammatory signaling of MAVS, such as NF-κB, interferon regulatory factor 3 (IRF3), and interferon α response, was repressed in *MAVS*^−/−^ hMSCs (Fig. S3A). Notably, the overall expression of senescence-associated secretory phenotype (SASP) factors, including interleukin 1α (IL1α), IL1β, IL6, IL8, CCL2, and CCL5, decreased in *MAVS*^−/−^ hMSCs (Fig. S3B and C). These observations are seemingly contradictory to the prevailing notion that increased SASP is not only the hallmark but also the key driver of many pathological changes of aging [18,49–53]. Given that the MAVS protein is localized in mitochondria and that mitochondrial dysfunction is associated with human stem cell senescence [14,18,45], we hypothesized that MAVS depletion-mediated hMSC senescence may result, at least partially, from impairment of mitochondrial fitness. Thus, we examined a series of parameters related to mitochondrial morphology and function in *MAVS*^+/+^ and *MAVS*^−/−^ hMSCs. We noticed that mitochondrial mass was increased in *MAVS*^−/−^ hMSCs at both late passage (LP) and even at early passage (EP) when the senescence phenotypes were not obviously manifested (Fig. 3A and Fig. S3D). In addition, electron microscopy analysis showed that elevated mitochondrial abundance, shrunken mitochondria, and more abnormal mitochondria were found in *MAVS*^−/−^ hMSCs compared with those in *MAVS*^+/+^ hMSCs (Fig. 3B and C). Consistently, mtDNA copy number was increased in *MAVS*^−/−^ hMSCs (Fig. 3D and Fig. S3E). Furthermore, we also observed compromised mitochondrial functions after MAVS depletion, demonstrated by decreased mitochondrial membrane potential, and increased mitochondrial ROS levels in *MAVS*^−/−^ hMSCs (Fig. 3E and F and Fig. S3F and G). Additionally, we observed a decline in the oxygen consumption rate (OCR) after MAVS depletion, as evidenced by decreased maximal respiration and spare respiratory capacity, even though equal levels of basal respiration and adenosine triphosphate (ATP) production were detected (Fig. 3G and Fig. S3H). These findings demonstrate that MAVS depletion compromises mitochondrial dynamics, leading to the destruction of mitochondrial structure and functional fitness.

### MAVS stabilizes OPA1 in hMSCs

**Fig. 4.**
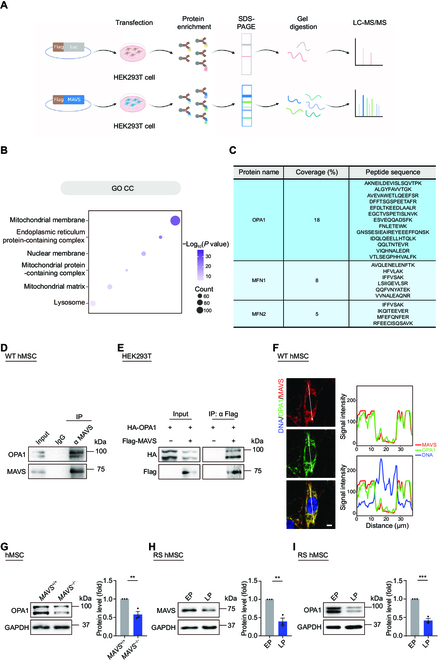
MAVS interacts with OPA1 in mitochondria in hMSCs. (A) Schematic diagram of the mass spectrometry strategy for identifying MAVS-interacting proteins. Flag-Luc was used as control. (B) Point plot showing enriched GO CC terms of MAVS-interacting proteins identified by mass spectrometry analysis. The color key from gray to purple indicates low to high enrichment levels. (C) OPA1, MFN1, and MFN2 were MAVS-interacting proteins identified by mass spectrometry. Peptide sequences of each interacting protein are listed in the table. (D) Co-IP analysis of endogenous OPA1 with MAVS protein in WT hMSCs. (E) Co-IP analysis of exogenous Flag-tagged MAVS protein with HA-tagged OPA1 protein in HEK293T cells. (F) Left: Immunofluorescence staining of OPA1 and MAVS in WT hMSCs at MP (P6). Right: Signal intensity analysis of OPA1 and MAVS. Scale bar, 10 μm. (G) Western blotting analysis of OPA1 in *MAVS*^+/+^ and *MAVS*^−/−^ hMSCs at LP (P8). GAPDH was used as a loading control. Data are presented as the means ± SEM. *n* = 3 biological replicates. **, *P* < 0.01 (*t* test). (H) Western blotting analysis of MAVS in RS WT hMSCs at EP (P3) and LP (P13). GAPDH was used as a loading control. Data are presented as the means ± SEM. *n* = 3 biological replicates. **, *P* < 0.01 (*t* test). (I) Western blotting analysis of OPA1 in RS WT hMSCs at EP (P4) and LP (P16). GAPDH was used as a loading control. Data are presented as the means ± SEM. *n* = 3 biological replicates. ***, *P* < 0.001 (*t* test).

To investigate the molecular mechanism by which MAVS regulates the mitochondrial activity and thereby hMSC homeostasis, we sought to identify potential MAVS interaction partners. Transfection of Flag-tagged MAVS was carried out in human embryonic kidney 293T (HEK293T) cells, followed by immunoprecipitation (IP) using an anti-Flag antibody (Fig. 4A). Then the resulting sample was subjected to liquid chromatography-tandem mass spectrometry (LC-MS/MS) analysis (Fig. 4A). Highly consistent with the mitochondrial localization of MAVS, GO Cellular Component (CC) analysis revealed that more than 10% of MAVS-interacting proteins are predicted to be mitochondrial membrane proteins (Fig. 4B). Likewise, according to the GO Biological Process (BP) analysis, MAVS-interacting proteins converge on the pathways related to mitochondrion organization (Fig. S4A). Among mitochondrial membrane proteins, we identified 3 proteins with a correspondingly high-affinity score that function in the mitochondrial fusion process, namely OPA1, mitofusin 1 (MFN1), and mitofusin 2 (MFN2) (Fig. 4C). The interactions between MAVS and MFN1 and MFN2 were consistent with previous studies [54–56]. Interestingly, we confirmed that mitochondrial fusion-associated protein OPA1 acted as a novel MAVS-interacting protein by performing an endogenous co-immunoprecipitation (Co-IP) assay (Fig. 4D). Additionally, the interaction of MAVS and OPA1 was also confirmed via exogenous Co-IP assay (Fig. 4E), and the colocalization of OPA1 and MAVS was verified via immunofluorescence staining in wild-type (WT) hMSCs (Fig. 4F).

Moreover, while the mRNA level of *OPA1* did not change, OPA1 protein abundance decreased in *MAVS*^−/−^ hMSCs relative to that in *MAVS*^+/+^ hMSCs (Fig. 4G and Fig. S4B), indicating that MAVS is required for the stable existence of OPA1 on mitochondrial membrane. Consistently, when we measured MAVS and OPA1 protein levels by western blotting in replicatively senescent (RS) WT hMSCs, we found that MAVS and OPA1 protein levels were reduced in RS hMSCs (Fig. 4H and I), confirming that deficiency of the MAVS–OPA1 axis is a critical molecular event associated with cellular senescence.

### Impaired OPA1 contributes to loss of mitochondrial homeostasis and cellular senescence

**Fig. 5.**
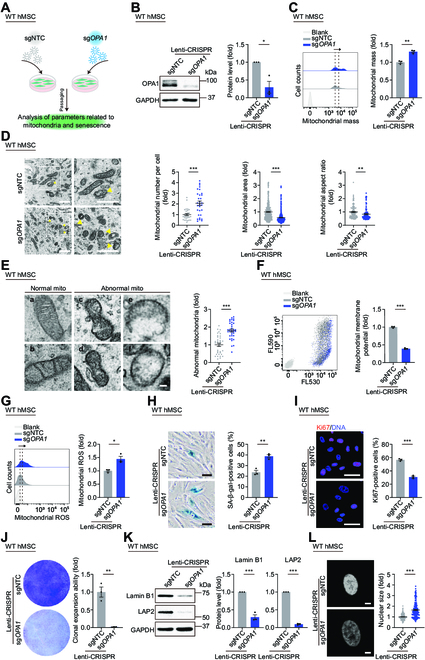
Impaired OPA1 contributes to the loss of mitochondrial homeostasis and cellular senescence. (A) Schematic workflow showing lentivirus infection mediating OPA1 knockdown in WT hMSCs at P7. (B) Western blotting analysis of OPA1 in WT hMSCs at P7 transduced with lentiviruses expressing sgNTC or sg*OPA1* via CRISPR/Cas9 system. GAPDH was used as a loading control. Data are presented as the means ± SEM. *n* = 3 biological replicates. *, *P* < 0.05 (*t* test). (C) FACS analysis of mitochondrial mass levels in WT hMSCs at P7 transduced with lentiviruses expressing sgNTC or sg*OPA1* via CRISPR/Cas9 system. Dashed lines indicate the position of MFI. Cells unstained were used as the blank control. Data are presented as the means ± SEM. *n* = 3 biological replicates. **, *P* < 0.01 (*t* test). (D) Left: TEM analyses of mitochondrial number, mitochondrial area and aspect ratio in WT hMSCs at P7 transduced with lentiviruses expressing sgNTC or sg*OPA1* via CRISPR/Cas9 system. Yellow arrows indicate abnormal mitochondria. Scale bar, 1 μm. Right: Statistical analyses of mitochondrial number per intact cell, mitochondrial area, and aspect ratio. Data are presented as the means ± SEM. *n* = 30 cells; *n* = 300 mitochondria; *n* = 100 mitochondria. **, *P* < 0.01; ***, *P* < 0.001 (*t* test). (E) Left: Micrographs of normal (a and b) and abnormal (c to f) mitochondria (“mito” indicates mitochondria). Scale bar, 100 nm. Right: Statistical analysis of relative percentage of abnormal mitochondria. Data are presented as the means ± SEM. *n* = 35 images to determine the relative percentage of abnormal mitochondria in each sample. ***, *P* < 0.001 (*t* test). (F) FACS analysis of mitochondrial membrane potential in WT hMSCs at P7 transduced with lentiviruses expressing sgNTC or sg*OPA1* via CRISPR/Cas9 system using a fluorescence probe JC-10. Cells unstained were used as the blank control. Data are presented as the means ± SEM. *n* = 3 biological replicates. ***, *P* < 0.001 (*t* test). (G) FACS analysis of mitochondrial ROS levels by MitoSOX red staining in WT hMSCs at P7 transduced with lentiviruses expressing sgNTC or sg*OPA1* via CRISPR/Cas9 system. Dashed lines indicate the position of MFI. Cells unstained were used as the blank control. Data are presented as the means ± SEM. *n* = 3 biological replicates. *, *P* < 0.05 (*t* test). (H) SA-β-gal staining in WT hMSCs at P7 transduced with lentiviruses expressing sgNTC or sg*OPA1* via CRISPR/Cas9 system. Scale bar, 50 μm. Data are presented as the means ± SEM. *n* = 3 biological replicates. **, *P* < 0.01 (*t* test). (I) Immunofluorescence staining of Ki67 in WT hMSCs at P7 transduced with lentiviruses expressing sgNTC or sg*OPA1* via CRISPR/Cas9 system. Scale bar, 50 μm. Data are presented as the means ± SEM. *n* = 3 biological replicates. ***, *P* < 0.001 (*t* test). (J) Clonal expansion analysis in WT hMSCs at P7 transduced with lentiviruses expressing sgNTC or sg*OPA1* via CRISPR/Cas9 system. Data are presented as the means ± SEM. *n* = 3 biological replicates. **, *P* < 0.01 (*t* test). (K) Western blotting analysis of aging-related markers Lamin B1 and LAP2 in WT hMSCs at P7 transduced with lentiviruses expressing sgNTC or sg*OPA1* via CRISPR/Cas9 system. GAPDH was used as a loading control. Data are presented as the means ± SEM. *n* = 3 biological replicates. ***, *P* < 0.001 (*t* test). (L) Left: Nuclear DNA staining in WT hMSCs at P7 transduced with lentiviruses expressing sgNTC or sg*OPA1* via CRISPR/Cas9 system. Right: Statistical analysis of nuclear size. Scale bar, 10 μm. Data are presented as the means ± SEM. *n* = 180 cells. ***, *P* < 0.001 (*t* test).

To further investigate the role of OPA1 in regulating mitochondrial homeostasis and hMSC senescence, we knocked down OPA1 in WT hMSCs using lentiviral CRISPR/Cas9-mediated gene editing (Fig. 5A) and validated the decreased protein level of OPA1 by western blotting analysis (Fig. 5B). OPA1 deficiency gave rise to dysregulated mitochondrial homeostasis in hMSCs, characterized by increased mitochondrial mass and abnormal mitochondrial morphology including increased mitochondrial abundance, shrunken mitochondria, and more abnormal mitochondria (Fig. 5C to E). We also observed mitochondrial dysfunction upon OPA1 knockdown, such as impaired mitochondrial membrane potential and accumulation of mitochondrial ROS (Fig. 5F and G). These phenotypes recapitulated the compromised mitochondrial homeostasis in *MAVS*^−/−^ hMSCs.

Next, we wondered whether OPA1 depletion caused hMSC senescence. Similar to our observations in *MAVS*^−/−^ hMSCs, we found accelerated senescence phenotypes in hMSCs upon OPA1 deficiency, including an increased percentage of SA-β-gal-positive cells, decreased percentage of Ki67-positive cells, and reduced colony formation ability (Fig. 5H to J). Knockdown of OPA1 was also associated with decreased Lamin B1 and LAP2, along with enlarged nuclear size (Fig. 5K and L and Fig. S4C). These data indicate that similar to that of MAVS, OPA1 depletion disrupts mitochondrial fitness and thereby induces hMSC senescence.

### Replenishment of MAVS alleviates mitochondrial dysfunction and senescence phenotypes in *MAVS*^−/−^ hMSCs

**Fig. 6.**
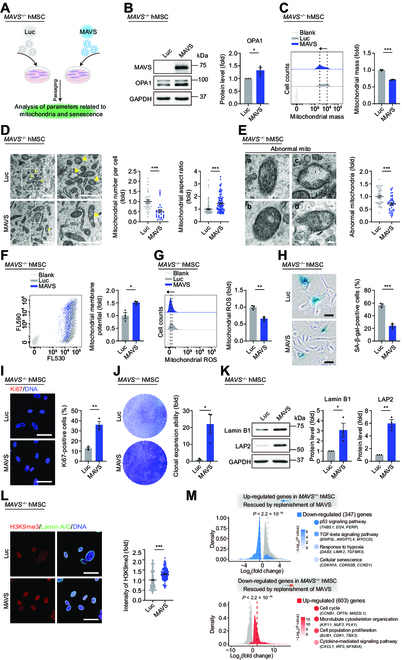
Replenishment of MAVS alleviates mitochondrial dysfunction and senescence phenotypes in *MAVS*^−/−^ hMSCs. (A) Schematic workflow showing lentivirus infection expressing Luc or MAVS in *MAVS*^−/−^ hMSCs at P5. (B) Western blotting analysis of MAVS and OPA1 in *MAVS*^−/−^ hMSCs at P5 transduced with lentiviruses expressing Luc or MAVS. Data are presented as the means ± SEM. *n* = 3 biological replicates. *, *P* < 0.05 (*t* test). (C) FACS analysis of mitochondrial mass levels in *MAVS*^−/−^ hMSCs at P5 transduced with lentiviruses expressing Luc or MAVS. Dashed lines indicate the position of MFI. Cells unstained were used as the blank control. Data are presented as the means ± SEM. *n* = 3 biological replicates. ***, *P* < 0.001 (*t* test). (D) Left: TEM analyses of mitochondrial number and aspect ratio in *MAVS*^−/−^ hMSCs at P5 transduced with lentiviruses expressing Luc or MAVS. Yellow arrows indicate abnormal mitochondria. Scale bar, 1 μm. Right: Statistical analyses of mitochondrial number per intact cell and mitochondrial aspect ratio. The statistical analysis of the mitochondrial area is shown in Fig. S5A. Data are presented as the means ± SEM. *n* = 30 cells; *n* = 100 mitochondria. ***, *P* < 0.001 (*t* test). (E) Left: Micrographs of abnormal (a to d) mitochondria (“mito” indicates mitochondria). Scale bar, 100 nm. Right: Statistical analysis of relative percentage of abnormal mitochondria. Data are presented as the means ± SEM. *n* = 40 images to determine the relative percentage of abnormal mitochondria in each sample. ***, *P* < 0.001 (*t* test). (F) FACS analysis of mitochondrial membrane potential in *MAVS*^−/−^ hMSCs at P5 transduced with lentiviruses expressing Luc or MAVS. Cells unstained were used as the blank control. Data are presented as the means ± SEM. *n* = 3 biological replicates. *, *P* < 0.05 (*t* test). (G) FACS analysis of mitochondrial ROS levels by MitoSOX red staining in *MAVS*^−/−^ hMSCs at P5 transduced with lentiviruses expressing Luc or MAVS. Dashed lines indicate the position of MFI. Cells unstained were used as the blank control. Data are presented as the means ± SEM. *n* = 3 biological replicates. **, *P* < 0.01 (*t* test). (H) SA-β-gal staining in *MAVS*^−/−^ hMSCs at P5 transduced with lentiviruses expressing Luc or MAVS. Scale bar, 50 μm. Data are presented as the means ± SEM. *n* = 3 biological replicates. ***, *P* < 0.001 (*t* test). (I) Immunofluorescence staining of Ki67 in *MAVS*^−/−^ hMSCs at P5 transduced with lentiviruses expressing Luc or MAVS. Scale bar, 50 μm. Data are presented as the means ± SEM. *n* = 3 biological replicates. **, *P* < 0.01 (*t* test). (J) Clonal expansion analysis in *MAVS*^−/−^ hMSCs at P5 transduced with lentiviruses expressing Luc or MAVS. Data are presented as the means ± SEM. *n* = 3 biological replicates. *, *P* < 0.05 (*t* test). (K) Western blotting analysis of aging-related markers Lamin B1 and LAP2 in *MAVS*^−/−^ hMSCs at P5 transduced with lentiviruses expressing Luc or MAVS. Data are presented as the means ± SEM. *n* = 3 biological replicates. *, *P* < 0.05; **, *P* < 0.01 (*t* test). (L) Left: Immunofluorescence staining of H3K9me3 and Lamin A/C in *MAVS*^−/−^ hMSCs at P5 transduced with lentiviruses expressing Luc or MAVS. Right: Statistical analysis of mean fluorescence intensity of H3K9me3. Scale bar, 50 μm. Data are presented as the means ± SEM. *n* = 140 cells. ***, *P* < 0.001 (*t* test). (M) Left: Density plot showing DEGs in *MAVS*^−/−^ hMSCs compared to *MAVS*^+/+^ hMSCs that were restored upon re-expression of MAVS. Two-sided Wilcoxon signed-rank test. Right: Point plot showing GO terms and pathways enriched by up-regulated (red) and down-regulated (blue) DEGs. The color keys from gray to red or blue indicate low to high enrichment levels.

To investigate a potential geroprotective role of MAVS in hMSCs, we reintroduced MAVS into *MAVS*^−/−^ hMSCs via lentiviral vector-mediated gene transfer (Fig. 6A and B) and revealed that the re-expression of MAVS in *MAVS*^−/−^ hMSCs rescued OPA1 protein level (Fig. 6B). To explore whether MAVS replenishment could restore mitochondrial functions, we examined mitochondrial parameters and observed that the re-expression of MAVS dampened mitochondrial mass (Fig. 6C). When performed electron microscopy analysis of *MAVS*^−/−^ hMSCs after re-expression of MAVS, we detected ameliorated mitochondrial morphology, including decreased mitochondrial abundance but expanding mitochondrial area and aspect ratio, as well as fewer abnormalities (Fig. 6D and E and Fig. S5A). In addition, lower mtDNA copy number, intensified mitochondrial membrane potential, and decreased mitochondrial ROS were detected in *MAVS*^−/−^ hMSCs after the reintroduction of MAVS (Fig. 6F and G and Fig. S5B). Furthermore, we observed a reduced percentage of SA-β-gal-positive cells, an increased percentage of Ki67-positive cells, and augmented colony formation ability after MAVS replenishment (Fig. 6H to J). MAVS re-expression alleviated levels of other senescence markers in *MAVS*^−/−^ hMSCs, as revealed by the observation that Lamin B1 and LAP2 were rescued at both protein and transcriptional levels (Fig. 6K and Fig. S5C and D). We also detected that re-expression of MAVS enhanced H3K9me3 fluorescence intensity and weakened the DNA damage degree as manifested by 53BP1 and γH2AX-double positive cells (Fig. 6L and Fig. S5E). The nuclear size was also diminished after the re-expression of MAVS (Fig. S5F). In addition, the transcriptomic analysis revealed that pathways related to cell cycle and DNA repair were elevated, while pathways related to p53 signaling and cellular senescence were down-regulated in *MAVS*^−/−^ hMSCs after re-expression of MAVS (Fig. S5G and H). Upon combined analysis of DEGs in *MAVS*^+/+^ and *MAVS*^−/−^ hMSCs, we found that MAVS re-expression restored the general alterations in transcriptional profiling of *MAVS*^−/−^ hMSCs (Fig. 6M and Fig. S5I and J). Our findings reveal that MAVS exerts a geroprotective role in hMSCs, likely at least partially attributed to its role in stabilizing mitochondrial OPA1 and maintaining mitochondrial fitness.

### Overexpression of OPA1 alleviates mitochondrial dysfunction and senescence in *MAVS*^−/−^ hMSCs

**Fig. 7.**
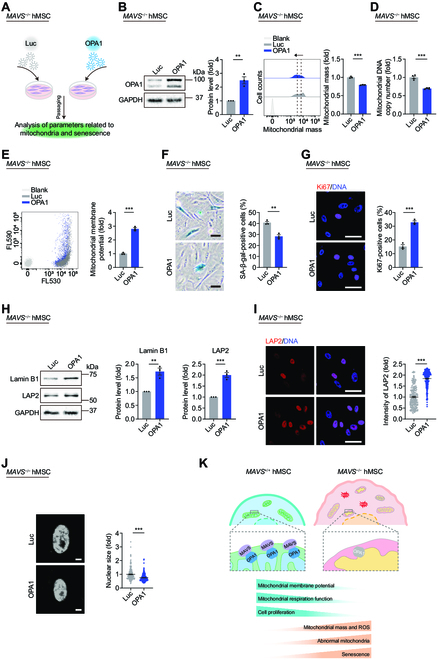
Overexpression of OPA1 alleviates mitochondrial dysfunction and senescence in *MAVS*^−/−^ hMSCs. (A) Schematic workflow showing lentivirus infection expressing Luc or OPA1 in *MAVS*^−/−^ hMSCs at P5. (B) Western blotting analysis of OPA1 in *MAVS*^−/−^ hMSCs at P5 transduced with lentiviruses expressing Luc or OPA1. Data are presented as the means ± SEM. *n* = 3 biological replicates. **, *P* < 0.01 (*t* test). (C) FACS analysis of mitochondrial mass levels in *MAVS*^−/−^ hMSCs at P5 transduced with lentiviruses expressing Luc or OPA1. Dashed lines indicate the position of MFI. Cells unstained were used as the blank control. Data are presented as the means ± SEM. *n* = 3 biological replicates. ***, *P* < 0.001 (*t* test). (D) qPCR analysis of mtDNA copy number in *MAVS*^−/−^ hMSCs at P5 transduced with lentiviruses expressing Luc or OPA1. Data are presented as the means ± SEM. *n* = 4 technological replicates. ***, *P* < 0.001 (*t* test). (E) FACS analysis of mitochondrial membrane potential in *MAVS*^−/−^ hMSCs at P5 transduced with lentiviruses expressing Luc or OPA1. Cells unstained were used as the blank control. Data are presented as the means ± SEM. *n* = 3 biological replicates. ***, *P* < 0.001 (*t* test). (F) SA-β-gal staining in *MAVS*^−/−^ hMSCs at P5 transduced with lentiviruses expressing Luc or OPA1. Scale bar, 50 μm. Data are presented as the means ± SEM. *n* = 3 biological replicates. **, *P* < 0.01 (*t* test). (G) Immunofluorescence staining of Ki67 in *MAVS*^−/−^ hMSCs at P5 transduced with lentiviruses expressing Luc or OPA1. Scale bar, 50 μm. Data are presented as the means ± SEM. *n* = 3 biological replicates. ***, *P* < 0.001 (*t* test). (H) Western blotting analysis of aging-related markers Lamin B1 and LAP2 in *MAVS*^−/−^ hMSCs at P5 transduced with lentiviruses expressing Luc or OPA1. Data are presented as the means ± SEM. *n* = 3 biological replicates. **, *P* < 0.01; ***, *P* < 0.001 (*t* test). (I) Left: Immunofluorescence staining of LAP2 in *MAVS*^−/−^ hMSCs at P5 transduced with lentiviruses expressing Luc or OPA1. Right: Statistical analysis of mean fluorescence intensity of LAP2. Scale bar, 50 μm. Data are presented as the means ± SEM. *n* = 220 cells. ***, *P* < 0.001 (*t* test). (J) Left: Nuclear DNA staining in *MAVS*^−/−^ hMSCs at P5 transduced with lentiviruses expressing Luc or OPA1. Right: Statistical analysis of nuclear size. Scale bar, 10 μm. Data are presented as the means ± SEM. *n* = 220 cells. ***, *P* < 0.001 (*t* test). (K) A model illustrating that the MAVS–OPA1 axis sustains mitochondrial homeostasis and antagonizes cellular senescence. In *MAVS*^+/+^ hMSCs, MAVS functions as a scaffold to maintain OPA1, thereby ensuring mitochondrial fitness and cellular homeostasis. In *MAVS*^−/−^ hMSCs, MAVS deletion leads to increased OPA1 instability on mitochondria, thus resulting in mitochondrial dysfunction and cellular senescence.

To further confirm that OPA1 functions as a downstream target of MAVS, we overexpressed OPA1 in *MAVS*^−/−^ hMSCs using a lentiviral vector (Fig. 7A and B). After OPA1 overexpression, we observed that mitochondrial dysfunction and senescence phenotypes in *MAVS*^−/−^ hMSCs were alleviated, as evidenced by reduced mitochondrial mass and mtDNA copy number and rescued mitochondrial membrane potential (Fig. 7C to E). More importantly, elevated OPA1 expression also reduced the percentage of SA-β-gal-positive cells, improved the percentage of Ki67-positive cells, and up-regulated protein levels of Lamin B1 and LAP2 in *MAVS*^−/−^ hMSCs (Fig. 7F to I). In addition, the smaller nuclear size was also observed after overexpression of OPA1 in *MAVS*^−/−^ hMSCs (Fig. 7J). These data further demonstrate that the MAVS–OPA1 axis plays an important role in regulating mitochondrial homeostasis and cellular senescence and portend MAVS and OPA1 as potential targets for alleviating cellular senescence (Fig. 7K).

## Discussion

In this study, we discovered a noncanonical geroprotective role for MAVS in maintaining mitochondrial fitness in hMSCs. First, we observed that MAVS depletion accelerated hMSC senescence. Second, MAVS deficiency impaired mitochondrial dynamics, as reflected by increased fragmented mitochondria, accumulated mtDNA copy number, and increased mitochondrial mass, accompanied by decreased respiratory capacity and mitochondrial membrane potential and increased levels of ROS. Mechanistically, we found that MAVS interacted with and stabilized OPA1, a guanosine triphosphatase localized on the mitochondrial membrane, thereby maintaining mitochondrial homeostasis. In addition, OPA1 deficiency led to dysfunction of mitochondria and cellular senescence; however, replenishment of MAVS or OPA1 alleviated mitochondrial dysfunction and senescence phenotypes in *MAVS*^−/−^ hMSCs. Thus, to our knowledge, our findings reveal a novel role for MAVS in alleviating human stem cell senescence through modulating mitochondrial homeostasis.

Using a stem cell-based senescence model, we here, for the first time, revealed that MAVS maintained mitochondrial dynamics, thereby preventing stem cells from entering senescence. Mitochondrial dysfunction, characterized by swollen mitochondria, fragmentation of the mitochondrial network, and imbalance of mitochondrial functions, is a hallmark of aging [18,57–59]. This phenomenon has been observed in aged flies, worms, and human stem cells [16,45,60–64]. Our study revealed that MAVS depletion impaired mitochondrial dynamics and homeostasis, as reflected by abnormal mitochondrial morphology and compromised mitochondrial functions. We also identified that MAVS functioned as a scaffold to stabilize mitochondrial OPA1, while depletion of OPA1 phenocopied the mitochondrial dysfunction observed in *MAVS*^−/−^ hMSCs. In support of our results, related studies report that MAVS plays a role in the turnover of mitochondria via autophagy and in regulating mitochondrial metabolic homeostasis via interacting with voltage-dependent anion channel 2 [65,66]. In addition to OPA1, our LC-MS/MS analysis identified a number of proteins that maintain mitochondrial homeostasis as interacting partners for MAVS. The fine regulation of different aspects of mitochondria by MAVS deserves further study.

In line with a classical role of MAVS in the antiviral innate immune response, our findings demonstrated that its depletion led to reduced levels of inflammatory cytokines. Although it is not known how mitochondrial localized MAVS coordinates the control of mitochondrial integrity and antiviral responses, there is growing evidence that mitochondrial proteins are involved in regulating the antiviral activity of MAVS. For example, the mitochondrial fusion protein MFN1 may bind to MAVS and positively regulate the MAVS-mediated antiviral response [56], while mitofusin protein MFN2 interacts with MAVS and negatively regulates antiviral signaling [54,67]. Further study is demanded to demonstrate the multifaceted roles of MAVS in maintaining cellular homeostasis.

Our study shows that the mitochondrial fusion protein OPA1 associated with MAVS plays an important role in antagonizing cellular senescence. Consistently, several studies have revealed the participation of mitochondrial-fusion-related processes during aging and age-related disease. Disruption of mitochondrial fusion by knockdown of Mfn1 or Mfn2 was reported to cause mouse NSC exhaustion, and mutation of the *MFN2* gene led to Charcot-Marie-Tooth type 2A disorder [30,68]. In addition, age-related decline of OPA1 was associated with muscle loss, and acute muscle-specific deletion of *Opa1* caused precocious senescence phenotypes and premature death in mice [69]. In aggregate, these studies demonstrate the important roles of mitochondria dynamics in cellular senescence and organ aging. Although the mechanism by which MAVS knockout does not induce aging in hESC is unclear, one may attribute this effect to the strong tolerance of pluripotent stem cells to genetic defects [70].

Here, we elucidate a distinct role for endogenous MAVS in regulating mitochondrial homeostasis and senescence in hMSCs. Further investigation is demanded to fully comprehend the complex regulatory mechanisms that control MAVS expression, and its impact on the interplay between antiviral response and mitochondrial dynamics that contributes to the aging process.

## Materials and Methods

### Animal experiments

The formation of teratoma and the hMSC transplantation assays in vivo were conducted as previously reported [44].

For teratoma formation assay, *MAVS*^+/+^ and *MAVS*^−/−^ hESCs were cultured on plates precoated with Matrigel (BD Biosciences). Cells were then collected in a Matrigel/mTeSR (STEMCELL Technologies, Vancouver) mixture, and subsequently transplanted subcutaneously into male NOD/SCID mice (6 to 8 wk old). Approximately 10 weeks after transplantation, teratomas derived from hESCs were collected for staining analysis. The expressions of FOXA2, SMA, and TuJ1, typical markers for the 3 germ layers, including endoderm, mesoderm, and ectoderm, respectively, were detected.

For in vivo hMSC transplantation assay, male nude mice (6 to 8 wk old) were injected with approximately 1 × 10^6^ of *MAVS*^+/+^ or *MAVS*^−/−^ hMSCs transfected with luciferase-expressing vector, into the TA muscle. The in vivo imaging system was employed to analyze the luciferase activity according to previous studies [42,45].

All animal experiments were authorized by the Chinese Academy of Sciences Institutional Animal Care and Use Committee. Mice were purchased from SiPeiFu (Beijing) Biotechnology Co., Ltd and raised with a standard laboratory diet under conditions of 25 °C and a 12-h light-dark cycle. Isoflurane or cervical dislocation was used for anesthesia or euthanasia, respectively.

### Cell culture

*MAVS*^+/+^ hESCs (derived from Line H9, WiCell Research Institute) and *MAVS*^−/−^ hESCs grown on mouse embryonic fibroblast (MEF) feeder layers inactivated with mitomycin C were cultured with conventional DMEM/F12 (Gibco) culture medium [42]. hESCs were also maintained on precoated plates with Matrigel (BD Biosciences) using mTeSR medium. hESC-derived hMSCs were maintained with hMSC culture medium [43]. hNSCs were cultured according to a previous study [71].

### Generation of *MAVS*^−/−^ hESCs

Gene editing was performed using CRISPR/Cas9 system according to previous studies [42,45]. The pCAG-mCherry-gRNA vector (Addgene, #87110) was used to clone the sgRNA targeting the *MAVS* gene in exon 2. Subsequently, H9 hESCs were electroporated with both the sgRNA vector and pCAG-1BPNLS-Cas9-1BPNLS-2AGFP (Addgene, #87109) using 4D-Nucleofector (Lonza) at a mass ratio of 1:2. After cells were cultured on 6-well plates precoated with Matrigel in mTeSR medium for 48 h, FACS system (BD FACSAria) was applied to sort green fluorescent protein-mCherry-double positive cells, and MEF feeders were used to culture the sorted cells in hESC culture medium. Genomic DNA extraction and PCR analysis, followed by sequencing, were performed from emerging hESC clones. Table S1 contains a list of sgRNA sequences and primers used for targeting the *MAVS* locus.

### Generation and characterization of *MAVS*^+/+^ and *MAVS*^−/−^ hMSCs

The *MAVS*^+/+^ and *MAVS*^−/−^ hESCs were differentiated into hMSCs according to previous studies [44,72]. Briefly, after approximately 3 d of culture on MEF feeders, *MAVS*^+/+^ and *MAVS*^−/−^ hESCs were digested with Dispase. The cells were then plated on low-adhesion 6-well plates for 72 h to obtain embryoid bodies, which were subsequently transferred into precoated 6-well plates with Matrigel for differentiation with hMSC culture medium. The emerged fibroblast-like cells were passaged into a new 6-well plate precoated with gelatin (Sigma-Aldrich) in hMSC culture medium for continued cultivation after around 10 d. After the cells reached about 90% confluence, they were harvested and purified by selecting hMSC-specific markers including CD105, CD73, and CD90, using a FACS system. CD14, CD19, and CD34 as negative hMSC markers were also analyzed by FACS to confirm the purity of hMSCs. The pluripotent capacity of hMSCs was evaluated by trilineage differentiation [73,74].

### Generation and characterization of *MAVS*^+/+^ and *MAVS*^−/−^ hNSCs

The differentiation of *MAVS*^+/+^ and *MAVS*^−/−^ hESCs into hNSCs was described previously [71]. H9-hESCs were picked and plated onto 6-well plates preseeded with MEF feeder cells in hESC culture medium for about 2 d. The medium was subsequently substituted with hNSC differentiation medium. Then, the cells were passaged onto plates precoated with Matrigel after digestion with Accumax (Millipore) and maintained in hNSC culture medium.

### Whole-genome sequencing and CNV analysis

Genomic DNA extraction was conducted according to previous studies [42,44]. The quality control, library construction, and sequencing were carried out by Novogene Bioinformatics Technology Co., Ltd according to the instructions of the manufacturer. CNV analysis was executed as previously mentioned [75].

To perform CNV analysis, the raw reads were first trimmed using Trim Galore (version 0.4.5), followed by alignment to the human hg19 genome with Bowtie2 (version 2.2.9) [76]. The resulting reads were calculated in each 500-kb bin using the “readCounter” function from the hmmcopy_utils (https://github.com/shahcompbio/hmmcopy_utils) tool. To correct the GC content, copy number, and mappability, R/Bioconductor package HMMcopy (version 1.26.0) was employed.

### Genotype analysis and off-target detection in *MAVS*^−/−^ hESCs

Genomic DNA was extracted using a DNA Extraction Kit (TIANGEN). For genotyping of *MAVS*^−/−^ hESC clones, DNA fragments containing sgRNA targets were amplified with primers and cloned into the empty pEASY vector using a cloning kit (TransGen) for sequencing. To detect off-target effects in *MAVS*^−/−^ hESCs, PCR amplification was performed on regions with high off-target potential using a PrimeSTAR DNA Polymerase Kit (TaKaRa), followed by sequencing of the purified PCR products using the QIAquick PCR Purification Kit (QIAGEN). Table S1 contains a list of primers used for PCR amplification.

### RNA extraction, quantitative reverse transcription-PCR (qRT-PCR), and RNA-seq

Total RNA extraction was conducted according to previous studies [42,44], followed by qRT-PCR analysis on a CFX384 Real-Time PCR Detection System (Bio-Rad) using THUNDERBIRD SYBR Green qPCR Mix (TOYOBO). Table S2 contains a list of primers used for qRT-PCR analysis.

For RNA-seq, a VAHTS Universal V6 RNA-seq Library Prep Kit for Illumina (NR604-01/02) was used for sequencing library construction with index codes added to capture sequences of each sample following the recommendations of the manufacturer. The Illumina NovaSeq 6000 platform was utilized for paired-end sequencing with read length of 150 bp. RNA quality control and sequencing were carried out by Annoroad Gene Technology Co., Ltd.

### Plasmid construction

To generate plasmids overexpressing MAVS, or OPA1, the coding sequence of each gene was amplified using complementary DNA as a template with a PrimeSTAR DNA Polymerase Kit and cloned into the pLE4 vector, which is a gift from Tomoaki Hishida [75] via the XbaI-SalI sites for MAVS and BamHI-SalI sites for OPA1. To generate hemagglutinin (HA)- or Flag-tagged MAVS- or OPA1-overexpressing plasmids, the coding sequences of HA or Flag were designed in the reverse primers of MAVS or OPA1 directly. Plasmids overexpressing luciferase (luc) were used as negative controls [75]. Table S3 contains a list of primers used in the experiment.

For CRISPR/Cas9-mediated OPA1 gene knockdown, the OPA1-specific sgRNA was cloned into lenti-CRISPRv2 (Addgene, #52961) via the Esp3I site. Table S3 contains the sequences of the sgRNA.

### Lentivirus packaging and cell infection

The designated lentiviral overexpression or knockdown plasmids together with lentiviral packaging vectors including psPAX2 (Addgene, #12260) and pMD2.G (Addgene, #12259) were cotransfected into HEK293T cells using polyethylenimine (Polysciences). Lentiviral particles in the culture medium were harvested after 2 and 3 d and then purified through ultracentrifugation at 4 °C at 19,400 rpm for 2.25 h. For lentivirus transduction, WT hMSCs (P7) or *MAVS*^−/−^ hMSCs (P5) were seeded and then transfected with lentiviruses supplemented with polybrene (Sigma-Aldrich) for 24 h. To enrich lentivirus-transduced OPA1 knockdown, puromycin (1 μg/ml, Gibco) was added to cells at 48 h after infection. After several serial passages, further analyses were performed, including immunofluorescence staining, clonal expansion assay, SA-β-gal staining, and protein extraction.

### RNA-seq data processing

To process the RNA-seq raw data, low-quality reads and adaptor-containing sequences were eliminated using Trim Galore (version 0.4.5). The trimmed data were then aligned to the human hg19 reference genome using STAR software (version 2.7.1a) with default settings [77]. Gene expression levels were determined by counting the reads assigned to each gene using featureCounts (version 2.0.1) [78]. A criterion of absolute log_2_(fold change) over 0.5 and Benjamini–Hochberg adjusted *P* value less than 0.05 was used to obtain DEGs by R package DESeq2 (version 1.30.1) [79]. Metascape was applied to identify enriched GO terms and pathways [80]. Public gene sets from the Molecular Signatures Database (MSigDB) [81] and Regeneration Roadmap database [82] were acquired. Gene Set Enrichment Analysis (GSEA) software (version 4.1.0) was used to conduct gene set enrichment analysis with default parameters [83]. Table S4 contains a list of DEGs.

### Co-IP analysis

Co-IP assays were performed following previous studies [16,45]. MAVS-interacting proteins were identified through Co-IP using plasmids expressing Flag-Luc or Flag-MAVS transfected into HEK293T cells. After 48 h, the cells were collected and lysed in CHAPS buffer, which contained 0.3% CHAPS, 40 mM Hepes, 1 mM EDTA (pH 7.5), and 120 mM NaCl, supplemented with 1× complete protease and phosphatase inhibitor cocktail (Roche), and 1× phenylmethylsulfonyl fluoride (Dingguochangsheng Biotech). Following 2 h of rotation on a rotator at 4 °C, cell lysates were then centrifuged at a speed of 12,000 rpm for 30 min at 4 °C. The supernatants were collected and the protein concentrations were quantified using a BCA Kit (Dingguochangsheng Biotech). Equal amounts of protein were adjusted and incubated with beads (ANTI-FLAG M2 Affinity Gel, Sigma-Aldrich) coupled with anti-Flag antibody on a rotator at 4 °C for more than 8 h. Immunoprecipitates were spun down and then washed 5 times for 5 min each time. For competitive elution, the pellets were incubated with Flag peptides (Sigma-Aldrich) by rotating for 3 h at 4 °C. At last, the supernatants were collected, dissolved in 2× sodium dodecyl sulfate (SDS) loading buffer (Dingguochangsheng Biotech), boiled for 10 min at 95 °C, and then stored at −80 °C used for subsequent analysis.

For verifying interactions, the endogenous Co-IP assay was performed in WT hMSCs lysed with lysis buffer (0.5% NP-40, 50 mM tris-HCl [pH 8.0], and 120 mM NaCl) supplemented with 1× complete protease and phosphatase inhibitor cocktail. The supernatants (2 mg of protein) were then mixed with indicated antibodies rotated at 4 °C overnight and subsequently incubated with Protein A/G PLUS Agarose beads (Santa Cruz Biotechnology) for 2 h at 4 °C. The immunocomplexes were eluted by boiling. Regarding the exogenous Co-IP assay, HEK293T cells were transfected with the indicated plasmids and subsequently harvested 48 h after transfection and lysed using lysis buffer.

### LC-MS/MS analysis

The eluates obtained from Co-IP were subjected to 10% SDS-polyacrylamide gel electrophoresis (PAGE) for protein separation. Then Coomassie brilliant blue staining of the gel was performed for 1 h, and the gel was rinsed several times for decolorization with a destaining solution (methanol:glacial acetic acid:deionized water = 5:1:4). After molecular weight was determined, bands of interest were extracted from the gel and analyzed using LC-MS/MS. A Q Exactive mass spectrometer (Thermo Fisher Scientific) was used to acquire a high resolution of MS data at 70,000 (*m/z* 200) spanning the mass range of 300 to 1,600 *m/z*. Then, Proteome Discovery (version 2.2.0.388) with the Sequest HT search engine was used for protein identification from the raw data. The UniProt human protein database (updated on October 2017) was used to search for peptide data. Peptide filtering was performed with high confidence in protein identification; the high peptide confidence was set to ensure accuracy, and the false discovery rate analytical threshold was set at 1% in Percolator. MAVS-interacting proteins are listed in Table S5.

GO CC and GO BP enrichment analysis for MAVS-interacting proteins were performed by Metascape [80].

### Antibodies

The following antibodies were used for western blotting, immunofluorescence staining, and flow cytometry analysis, respectively: anti-MAVS (Santa Cruz Biotechnology, sc-166583, 1:1,000), anti-OPA1 (BD Biosciences, 612606, 1:1,000), anti-p21^Cip1^ (Cell Signaling Technology [CST], 2947S, 1:1,000), anti-Lamin B1 (Abcam, ab16048, 1:1,000), anti-LAP2 (BD Biosciences, 611000, 1:1,000), anti-Flag (Sigma-Aldrich, F1804, 1:2,000), anti-HA (CST, 3724S, 1:1,000), anti-glyceraldehyde-3-phosphate dehydrogenase (GAPDH) (Santa Cruz Biotechnology, sc-365062, 1:3,000), anti-OPA1 (CST, 67589S, 1:200), anti-TOM20 (Santa Cruz Biotechnology, sc-11415, 1:200), anti-NANOG (Abcam, ab21624, 1:100), anti-SOX2 (Santa Cruz Biotechnology, sc-17320, 1:200), anti-OCT3/4 (Santa Cruz Biotechnology, sc-5279, 1:200), anti-FOXA2 (CST, 8186S, 1:200), anti-SMA (ZSGB-BIO, ZM-0003, 1:200), anti-TuJ1 (Sigma-Aldrich, T2200, 1:200), anti-PAX6 (BioLegend, 901301, 1:200), anti-Nestin (BD Biosciences, 560422, 1:200), anti-Ki67 (ZSGB-BIO, ZM0166, 1:1,000), anti-LAP2 (BD Biosciences, 611000, 1:250), anti-H3K9me3 (Abcam, ab8898, 1:500), anti-Lamin A/C (Santa Cruz Biotechnology, sc-376248, 1:500), anti-53BP1 (Bethyl Laboratories, A300-273A, 1:200), anti-γH2AX (Millipore, 05-636, 1:200), anti-CD73-PE (BD Biosciences, 550257, 1:200), anti-CD90-FITC (BD Biosciences, 555595, 1:200), anti-CD105-APC (BD Biosciences, 17-1057-42, 1:200), anti-CD14-PE (BD Biosciences, 555398, 1:200), anti-CD19-APC (BD Biosciences, 555415, 1:200), anti-CD34-FITC (BD Biosciences, 555821, 1:200), HRP-conjugated Goat anti-Mouse IgG (H+L) (ZSGB-BIO, ZB-2305, 1:5000), HRP-conjugated Goat anti-Rabbit IgG (H+L) (ZSGB-BIO, ZB-2301, 1:5000), Alexa Fluor 488 Donkey anti-Mouse IgG (H+L) (Invitrogen, A21202, 1:500), Alexa Fluor 568 Donkey anti-Rabbit IgG (H+L) (Invitrogen, A10042, 1:500), and Alexa Fluor 647 Donkey anti-Goat IgG (H+L) (Invitrogen, A21447, 1:500).

### Western blotting

The cells were lysed with 1× SDS lysis buffer, containing 62.5 mM tris-HCl (pH 6.8) and 2% (wt/vol) SDS, and then boiled for 10 min at 95 °C. The BCA Kit was used to access the protein concentrations. The SDS-PAGE was performed as previously reported, and chemiluminescent imaging was carried out on a ChemiDoc XRS+ system (Bio-Rad Laboratories, Inc.) using the Image Lab software [84]. Protein band intensity was quantified using ImageJ.

### Immunofluorescence microscopy

Coverslips (Thermo Fisher Scientific) were used for seeding the cells. Until about 80% confluency, cells were subsequently followed by the routine immunofluorescence staining procedure [43]. After being washed twice with phosphate-buffered saline (PBS), cells were subjected to fixation for 15 min with 4% paraformaldehyde and permeabilization and blocking with Triton X-100 (0.2%, Sigma-Aldrich) for 10 min and donkey serum (10%, Jackson ImmunoResearch) for 1 h at room temperature, respectively. Following incubation with primary antibodies, the coverslips were incubated with secondary antibodies. Finally, Hoechst 33342 (Thermo Fisher Scientific) was used to label the nuclei. Zeiss Confocal System LSM900 and Leica SP5 Confocal System were applied to capture images.

### Transmission electron microscopy (TEM)

TEM was used to observe the fine structure of mitochondrion following previous studies [16,84,85]. Briefly, approximately 5 × 10^6^ cells per sample were harvested after trypsinization, washed with PBS, and centrifuged for 5 min at a speed of 1,000 rpm. Subsequently, fixation of cell pellets was performed using 2.5% (vol/vol) glutaraldehyde (brought to room temperature in advance) within phosphate buffer (0.1 M, pH 7.4) at room temperature for about 20 min, then overnight at 4 °C. Routine procedures for heavy metal staining were performed [84]. TEM (FEI Tecnai Spirit 120 kV) was employed to capture images.

Mitochondrial counting was performed in whole cells, and the data were derived from 30 to 40 cells in each sample. The area and aspect ratio of mitochondria for each sample were calculated from over 100 mitochondria. The definition and quantification of mitochondrial abnormality were based on previous studies [16,86,87], where abnormal mitochondria were described as obvious changes in mitochondrial matrix density, formation of membrane vesiculates, and disruption of mitochondrial cristae structure. The mitochondrial number, area, and aspect ratio were determined utilizing ImageJ.

### SA-β-gal staining assay

To assess the senescent cells, SA-β-gal staining was performed following previously published methods [88–90]. Briefly, fixation buffer containing 0.2% glutaraldehyde and 2% formaldehyde was used to fix cells for 5 min. The fresh SA-β-gal staining solution was prepared and used for staining at 37 °C in the dark for about 8 h. The optical microscope was applied to capture the images, and ImageJ was utilized to quantify the percentage of cells positive for SA-β-gal staining.

### Clonal expansion assay

The clonal expansion assay to assess the cell proliferation ability was conducted as previously described [74,75]. A scanner (EPSON, V370) was used to capture the images, and the ImageJ software was employed to calculate the density ratio of cells.

### Cell cycle analysis

Cells were digested using TrypLE and washed with PBS, then fixed by precooled 70% ethyl alcohol overnight. The next day, after centrifugation at 2,500 rpm at 4 °C, the supernatants from each tube were discarded, and cell pellets were resuspended and incubated with PBS solution containing ribonuclease A (0.2 mg/ml, TIANGEN, for degrading RNA), propidium iodide (0.02 mg/ml, Invitrogen) and Triton X-100 (0.1%) for 30 min at 37 °C. Flow cytometry (BD FACSCalibur) was used to perform the cell cycle measurement.

### Measurement of total cellular ROS level

hMSCs were incubated with 2.5 μM H2DCFDA (Invitrogen, #C6827), a type of ROS indicator at 37 °C for 20 min, for total cellular ROS measuring. An LSRFortessa cell analyzer (BD Biosciences) was used for the measurement, and the FlowJo software (TreeStar, Ashland, OR) was used to analyze the data.

### Telomere length analysis

Quantitative PCR (qPCR) analysis was applied to detect the telomere intensity as previously described [91]. Table S2 contains a list of primers used for detecting telomere intensity.

### Quantification of relative mtDNA copy number

The mtDNA copy number was estimated based on the previous descriptions with some modifications [45,92]. Briefly, the relative ratio between the copy number of a mitochondrial gene (tRNA^Leu(UUR)^) and a nuclear gene (β-2-microglobulin) was used to define the mtDNA content via qPCR analysis. Before mtDNA copy number measurement, templates were treated with ribonuclease A to remove RNA contamination. Table S2 contains a list of primers used for analyzing mtDNA copy number.

### Measurements of mitochondrial ROS and mass and membrane potential (MMP)

Mitochondrial ROS levels were evaluated using MitoSOX Red (2.5 μM, Invitrogen, #M36008) at 37 °C for 20 min. The mass of mitochondria was measured using nonyl acridine orange (10 μM, Invitrogen, #A1372) at 37 °C for 20 min. Additionally, the JC-10 Assay Kit (AAT Bioquest, #22801) was utilized to determine the MMP following the instructions of the manufacturer. Briefly, living cells were incubated with the 1× JC-10 working solution for 20 min at 37 °C. The MMP levels were defined by the ratio of JC-10 aggregate emission and monomer emission according to previous studies [93,94]. All measurements above were conducted using flow cytometry (BD LSRFortessa).

### Cell mitochondrial stress test

The role of mitochondrial respiration was assessed via the addition of different regulators, and the OCR was analyzed according to previous studies with some modifications [15,16]. Two days before the experiment, hMSCs were seeded at a density of 1.4 × 10^4^ cells per Seahorse culture microplate well on gelatin-coated plates. The Seahorse Analyzer was warmed up 1 d in advance.

The assay medium was then prepared by supplementing the basal medium with 2 mM glutamine, 1 mM sodium pyruvate, and 10 mM glucose. The cell culture microplate was incubated in a CO_2_-free incubator at 37 °C for 1 h and washed twice with the assay medium before incubation. The Seahorse XF sensor cartridge was precalibrated and then filled with mitochondrial respiration regulators rotenone/antimycin A (0.5 μM), oligomycin (1 μM), and FCCP (2 μM). The software “Wave” was used to conduct and analyze the experiment, and the values of OCR were finally normalized with cell number per well.

### Statistical analysis

PRISM software (version 8, GraphPad Software) was used to statistically evaluate the data. Results were presented as the means ± SEM. Statistical analysis was performed using the 2-tailed unpaired Student *t* test, and *P* values less than 0.05 were considered statistically significant (*), while *P* values less than 0.01 and *P* values less than 0.001 were considered extremely statistically significant (** and ***).

## Data Availability

The study’s sequencing data were submitted to the Genome Sequence Archive [95] in the National Genomics Data Center, China National Center for Bioinformation, Chinese Academy of Sciences, with accession number HRA004255. The LC-MS/MS data have been deposited via the iProX partner repository [96] to the ProteomeXchange Consortium (http://proteomecentral.proteomexchange.org) under accession number PXD041140. The DEGs identified in *MAVS*^−/−^ hMSCs have been deposited to the Aging Atlas database (https://ngdc.cncb.ac.cn/aging/index) [97].

## Supplementary Materials

Supplementary 1 Fig. S1. Characterization of *MAVS*-deficient hESCs. Fig. S2. Phenotypes and RNA-seq analyses of *MAVS*-deficient pluripotent stem cells. Fig. S3. *MAVS*^−/−^ hMSCs show mitochondrial dysfunction. Fig. S4. Impaired OPA1 promotes cellular senescence via interacting with MAVS. Fig. S5. Replenishment of MAVS alleviates senescence phenotypes in *MAVS*^−/−^ hMSCs. Table S1. Sequence information of sgRNA and primers used for gene editing, genotyping, and off-target detection. Table S2. Primers used for q(RT-)PCR analysis. Table S3. Primers and sgRNA sequences used for plasmid construction. Table S4. DEGs between *MAVS*^+/+^ and *MAVS*^−/−^ hESCs, hNSCs, and hMSCs, as well as DEGs between *MAVS*^−/−^ hMSCs transduced with lentiviruses expressing Luc or MAVS (MAVS vs. Luc). Table S5. The candidate MAVS-interacting proteins identified by mass spectrometry.
