# Supplementary material for: MAVS Antagonizes Human Stem Cell Senescence as a Mitochondrial Stabilizer
Source: Research (Wash D C). 2023 Jul 27;6:0192. doi: 10.34133/research.0192 (PMC10374246; doi:10.34133/research.0192)
Supplement: Supplementary 1 — Fig. S1. Characterization of MAVS-deficient hESCs. Fig. S2. Phenotypes and RNA-seq analyses of MAVS-deficient pluripotent stem cells. Fig. S3. MAVS−/− hMSCs show mitochondrial dysfunction. Fig. S4. Impaired OPA1 promotes cellular senescence via interacting with MAVS. Fig. S5. Replenishment of MAVS alleviates senescence phenotypes in MAVS−/− hMSCs. Table S1. Sequence information of sgRNA and primers used for gene editing, genotyping, and off-target detection. Table S2. Primers used for q(RT-)PCR analysis. Table S3. Primers and sgRNA sequences used for plasmid construction. Table S4. DEGs between MAVS+/+ and MAVS−/− hESCs, hNSCs, and hMSCs, as well as DEGs between MAVS−/− hMSCs transduced with lentiviruses expressing Luc or MAVS (MAVS vs. Luc). Table S5. The candidate MAVS-interacting proteins identified by mass spectrometry. [file research.0192.f1.zip › SM.docx]

# Supplementary Materials

**Fig. S1.** Characterization of *MAVS*-deficient hESCs.

**Fig. S2.** Phenotypes and RNA-seq analyses of *MAVS*-deficient pluripotent stem cells.

**Fig. S3.** *MAVS*^-/-^ hMSCs show mitochondrial dysfunction.

**Fig. S4.** Impaired OPA1 promotes cellular senescence via interacting with MAVS.

**Fig. S5.** Replenishment of MAVS alleviates senescence phenotypes in *MAVS*^-/-^ hMSCs.

**Table S1.** Sequence information of sgRNAs and primers used for gene editing, genotyping, and off-target detection.

**Table S2.** Primers used for q(RT-)PCR analysis.

**Table S3.** Primers used for plasmid construction.

**Table S4.** Differentially expressed genes (DEGs) between *MAVS*^+/+^ and *MAVS*^-/-^ hESCs, hNSCs, and hMSCs, as well as DEGs between *MAVS*^-/-^ hMSCs transduced with lentiviruses expressing Luc or MAVS (MAVS vs. Luc).

**Table S5.** The candidate MAVS-interacting proteins identified by mass spectrometry.

# Supplementary Figure Legends

# Fig. S1. Characterization of *MAVS*-deficient hESCs.

1. Off-target detection in *MAVS*^-/-^ hESCs.
2. Immunofluorescence staining of FOXA2, SMA, and TuJ1 to mark endoderm, mesoderm, and ectoderm of teratoma derived from *MAVS*^+/+^ and *MAVS*^-/-^ hESCs, respectively. Scale bar, 25 μm.
3. Karyotype analysis of *MAVS*^-/-^ hESCs.

# Fig. S2. Phenotypes and RNA-seq analyses of *MAVS*-deficient pluripotent stem cells.

1. Immunofluorescence staining of Ki67 in *MAVS*^+/+^ and *MAVS*^-/-^ hESCs. Scale bar, 50 μm. Data are presented as the means ± SEM. *n* = 3 biological replicates. ns, not significant (*t* test).
2. Immunofluorescence staining of Ki67 in *MAVS*^+/+^ and *MAVS*^-/-^ hNSCs. Scale bar, 50 μm. Data are presented as the means ± SEM. *n* = 3 biological replicates. ns, not significant (*t* test).
3. Bar plot showing cell cycle in *MAVS*^+/+^ and *MAVS*^-/-^ hNSCs by FACS analysis. Data are presented as the means ± SEM. *n* = 3 biological replicates. ns, not significant (*t* test).
4. Heatmap showing qRT-PCR analysis of indicated aging-related genes in *MAVS*^+/+^ and *MAVS*^-/-^ hMSCs at late passage (LP, P8). GAPDH was used as an internal control.
5. Statistical analysis of the levels of p21^Cip1^, Lamin B1, and LAP2 in *MAVS*^+/+^ and *MAVS*^-/-^ hMSCs at LP (P8) in Fig. 2G. Data are presented as the means ± SEM. *n* = 3 biological replicates. **, *P* < 0.01, ***, *P* < 0.001 (*t* test).
6. Left: Immunofluorescence staining of LAP2 in *MAVS*^+/+^ and *MAVS*^-/-^ hMSCs at LP (P8). Scale bar, 50 μm. Right: Statistical analysis of mean fluorescence intensity of LAP2. *n* = 156 cells. ***, *P* < 0.001 (*t* test).
7. Characterization of the differentiation potentials of *MAVS*^+/+^ and *MAVS*^-/-^ hMSCs at early passage (EP, P2) into osteoblasts, adipocytes, and chondrocytes evaluated by Von Kossa, Oil Red O and Toluidine blue staining, respectively. The relative Von Kossa-positive area, absorbance of Oil Red O and diameter of the chondrocyte sphere were quantified. Scale bars, 250 μm, 50 μm, and 250 μm. Data are presented as means ± SEM. *n* = 3, 4 and 8 biological replicates. *, *P* < 0.05, ***, *P* < 0.001 (*t* test).
8. Copy number variation (CNV) analysis in *MAVS*^+/+^ and *MAVS*^-/-^ hMSCs at EP (P2).
9. Heatmap showing differentially expressed genes (DEGs) between *MAVS*^+/+^ and *MAVS*^-/-^ hMSCs at LP (P8). The color key from blue to red indicates row z-score from low to high.
10. Violin plot showing expression levels of DEGs associated with cell cycle in *MAVS*^+/+^ and *MAVS*^-/-^ hESCs, hNSCs and hMSCs
11. Violin plot showing expression levels of DEGs associated with DNA repair in *MAVS*^+/+^ and *MAVS*^-/-^ hESCs, hNSCs and hMSCs.

# Fig. S3. *MAVS*^-/-^ hMSCs show the dysfunction of mitochondria.

1. Ridge plots showing downregulated NF-kappa B signaling pathway, IRF3 target genes and IFN alpha response in *MAVS*^-/-^ hMSCs at LP (P8).
2. GSEA plot showing downregulated SASP in *MAVS*^-/-^ hMSCs at LP (P8).
3. Heatmap showing qRT-PCR analysis of SASP-associated genes in *MAVS*^+/+^ and *MAVS*^-/-^ hMSCs at LP (P8). GAPDH was used as an internal control.
4. FACS analysis of mitochondrial mass levels in *MAVS*^+/+^ and *MAVS*^-/-^ hMSCs at EP (P3). Dashed lines indicate the position of mean fluorescent intensity (MFI). Cells without any treatment were used as the blank control. Data are presented as the means ± SEM. *n* = 3 biological replicates. ***, *P* < 0.001 (*t* test).
5. qPCR analysis of mitochondrial DNA copy number in *MAVS*^+/+^ and *MAVS*^-/-^ hMSCs at EP (P4). Data are presented as the means ± SEM. *n* = 4 technological replicates. ***, *P* < 0.001 (*t* test).
6. FACS analysis of mitochondrial membrane potential in *MAVS*^+/+^ and *MAVS*^-/-^ hMSCs at EP (P3) using a fluorescence probe JC-10. Cells without any treatment were used as the blank control. Data are presented as the means ± SEM. *n* = 3 biological replicates. *, *P* < 0.05 (*t* test).
7. FACS analysis of mitochondrial ROS levels by MitoSOX red staining in *MAVS*^+/+^ and *MAVS*^-/-^ hMSCs at EP (P3). Dashed lines indicate the position of MFI. Cells without any treatment were used as the blank control. Data are presented as the means ± SEM. *n* = 3 biological replicates. *, *P* < 0.05 (*t* test).
8. Detection of the OCR in *MAVS*^+/+^ and *MAVS*^-/-^ hMSCs at MP (P6) in response to indicated mitochondrial modulators by Seahorse analysis. Basal respiration and ATP production were calculated by the OCR values. Data are presented as the means ± SEM. *n* = 5 biological replicates. ns, not significant (*t* test).

# Fig. S4. Impaired OPA1 promotes cellular senescence via interacting with MAVS.

1. Network plot showing enriched Gene Ontology Biological Process (GO BP) pathways of MAVS-interacting proteins identified by mass spectrometry.
2. Representative tracks showing the expression level of *OPA1* in *MAVS*^+/+^ and *MAVS*^-/-^ hMSCs at LP (P8).
3. Left: Immunofluorescence staining of LAP2 in WT hMSCs at P7 transduced with lentiviruses expressing sgNTC or sg*OPA1* via CRISPR/Cas9 system. Right: Statistical analysis of mean fluorescence intensity of LAP2. Scale bar, 50 μm. Data are presented as the means ± SEM. *n* = 180 cells. ***, *P* < 0.001 (*t* test).

# Fig. S5. Replenishment of MAVS alleviates senescence phenotypes in *MAVS*^-/-^ hMSCs.

1. Statistical analysis of the mitochondrial area in *MAVS*^-/-^ hMSCs at P5 transduced with lentiviruses expressing luciferase (Luc) or MAVS in Fig. 6D. Data are presented as the means ± SEM. *n* = 300 mitochondria. ***, *P* < 0.001 (*t* test).
2. qPCR analysis of mitochondrial DNA copy number in *MAVS*^-/-^ hMSCs at P5 transduced with lentiviruses expressing Luc or MAVS. Data are presented as the means ± SEM. *n* = 4 technological replicates. ***, *P* < 0.001 (*t* test).
3. qRT-PCR analysis of *LMNB1* and *TMPO* in *MAVS*^-/-^ hMSCs at P5 transduced with lentiviruses expressing Luc or MAVS. Data are presented as the means ± SEM. *n* = 4 technological replicates. ***, *P* < 0.001 (*t* test).
4. Left: Immunofluorescence staining of LAP2 in *MAVS*^-/-^ hMSCs at P5 transduced with lentiviruses expressing Luc or MAVS. Right: Statistical analysis of mean fluorescence intensity of LAP2. Scale bar, 50 μm. Data are presented as the means ± SEM. *n* = 121 cells. ***, *P* < 0.001 (*t* test).
5. Immunofluorescence staining of 53BP1 and γH2AX in *MAVS*^-/-^ hMSCs at P5 transduced with lentiviruses expressing Luc or MAVS. Scale bar, 25 μm. Data are presented as the means ± SEM. *n* = 3 biological replicates. **, *P* < 0.01 (*t* test).
6. Left: Nuclear DNA staining in *MAVS*^-/-^ hMSCs at P5 transduced with lentiviruses expressing Luc or MAVS. Right: Statistical analysis of nuclear size. Scale bar, 10 μm. Data are presented as the means ± SEM. *n* = 180 cells. ***, *P* < 0.001 (*t* test).
7. Heatmap showing the Euclidean distance between replicates of RNA-seq in *MAVS*^-/-^ hMSCs at P5 with lentiviruses expressing Luc or MAVS. The color key of the Euclidean distance from blue to white indicates strong to weak correlation.
8. Left: Volcano plot showing DEGs in *MAVS*^-/-^ hMSCs at P5 transduced with lentiviruses expressing Luc or MAVS. Right: GO term and pathway enrichment analysis for upregulated (red) and downregulated (blue) DEGs.
9. Venn plot showing the numbers of DEGs in *MAVS*^-/-^ hMSCs compared to *MAVS*^+/+^ hMSCs that were restored upon re-expression of MAVS. Sankey plot showing DEGs in *MAVS*^-/-^ hMSCs compared to *MAVS*^+/+^ hMSCs that were restored upon re-expression of MAVS.
