# Supplementary material for: MAVS Antagonizes Human Stem Cell Senescence as a Mitochondrial Stabilizer
Source: Research (Wash D C). 2023 Jul 27;6:0192. doi: 10.34133/research.0192 (PMC10374246; doi:10.34133/research.0192)
Supplement: Supplementary 1 — Fig. S1. Characterization of MAVS-deficient hESCs. Fig. S2. Phenotypes and RNA-seq analyses of MAVS-deficient pluripotent stem cells. Fig. S3. MAVS−/− hMSCs show mitochondrial dysfunction. Fig. S4. Impaired OPA1 promotes cellular senescence via interacting with MAVS. Fig. S5. Replenishment of MAVS alleviates senescence phenotypes in MAVS−/− hMSCs. Table S1. Sequence information of sgRNA and primers used for gene editing, genotyping, and off-target detection. Table S2. Primers used for q(RT-)PCR analysis. Table S3. Primers and sgRNA sequences used for plasmid construction. Table S4. DEGs between MAVS+/+ and MAVS−/− hESCs, hNSCs, and hMSCs, as well as DEGs between MAVS−/− hMSCs transduced with lentiviruses expressing Luc or MAVS (MAVS vs. Luc). Table S5. The candidate MAVS-interacting proteins identified by mass spectrometry. [file research.0192.f1.zip › SM_images.pdf]

Figure S1

A

hESC

Potential off-target (OT) site	Sequence (5'-3')	Locus	Off-target
On-target	ATTGCGGCAGATATACTTAT	Chr. 20, 3854650 to 3854669	—
OT-#1	GGCAGATATACTTAT	Chr. 1, 46327641 to 46327655	NO
OT-#2	GGCAGATATACTTAT	Chr. 8, 122115328 to 122115342	NO
OT-#3	ATTGCGGCAGATATA	Chr. 9, 10122925 to 10122939	NO
OT-#4	GGCAGATATACTTAT	Chr. 17, 19726929 to 19726943	NO
OT-#5	GGCAGATATACTTAT	Chr. X, 56025526 to 56025540	NO

B

hESC-derived teratoma

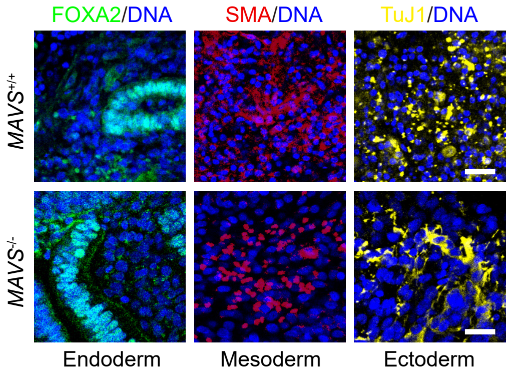

C

hESC

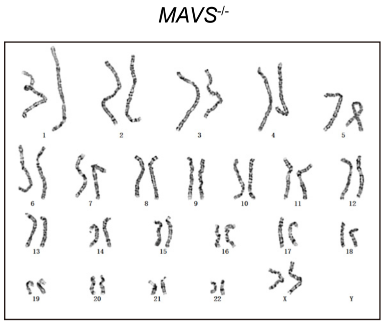

Figure S2

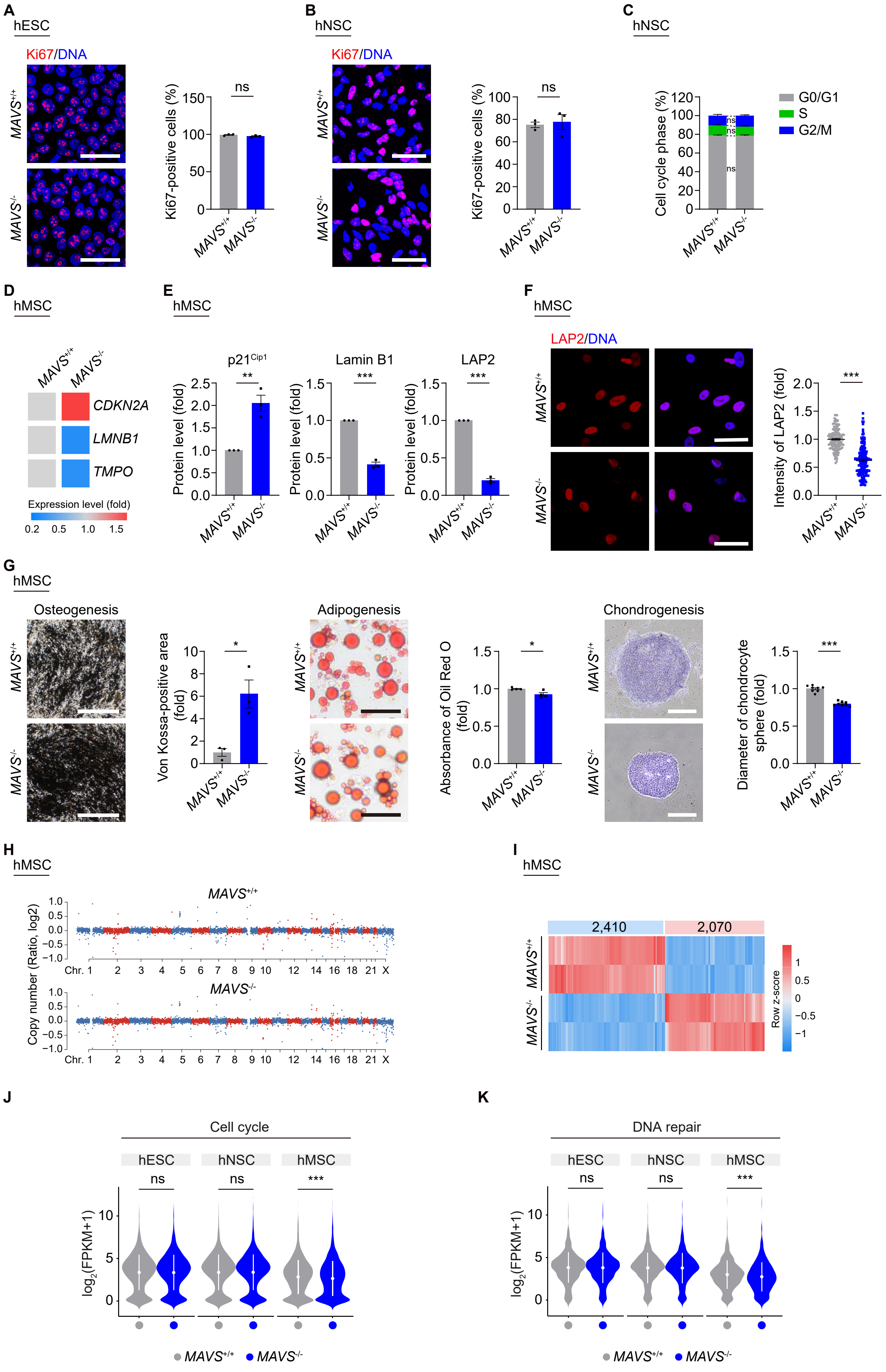

A
hESC
Ki67/DNA
MAVS+/+
MAVS-/-
Ki67-positive cells (%)
ns
B
hNSC
Ki67/DNA
C
hNSC
Cell cycle phase (%)
G0/G1
S
G2/M
D
hMSC
CDKN2A
LMNB1
TMPO
Expression level (fold)
E
hMSC
p21Cip1
Lamin B1
LAP2
Protein level (fold)
F
hMSC
LAP2/DNA
Intensity of LAP2 (fold)
G
hMSC
Osteogenesis
Von Kossa-positive area (fold)
Adipogenesis
Absorbance of Oil Red O (fold)
Chondrogenesis
Diameter of chondrocyte sphere (fold)
H
hMSC
Copy number (Ratio, log2)
I
hMSC
2,410
2,070
Row z-score
J
Cell cycle
hESC
hNSC
hMSC
log2(FPKM+1)
K
DNA repair

Figure S3

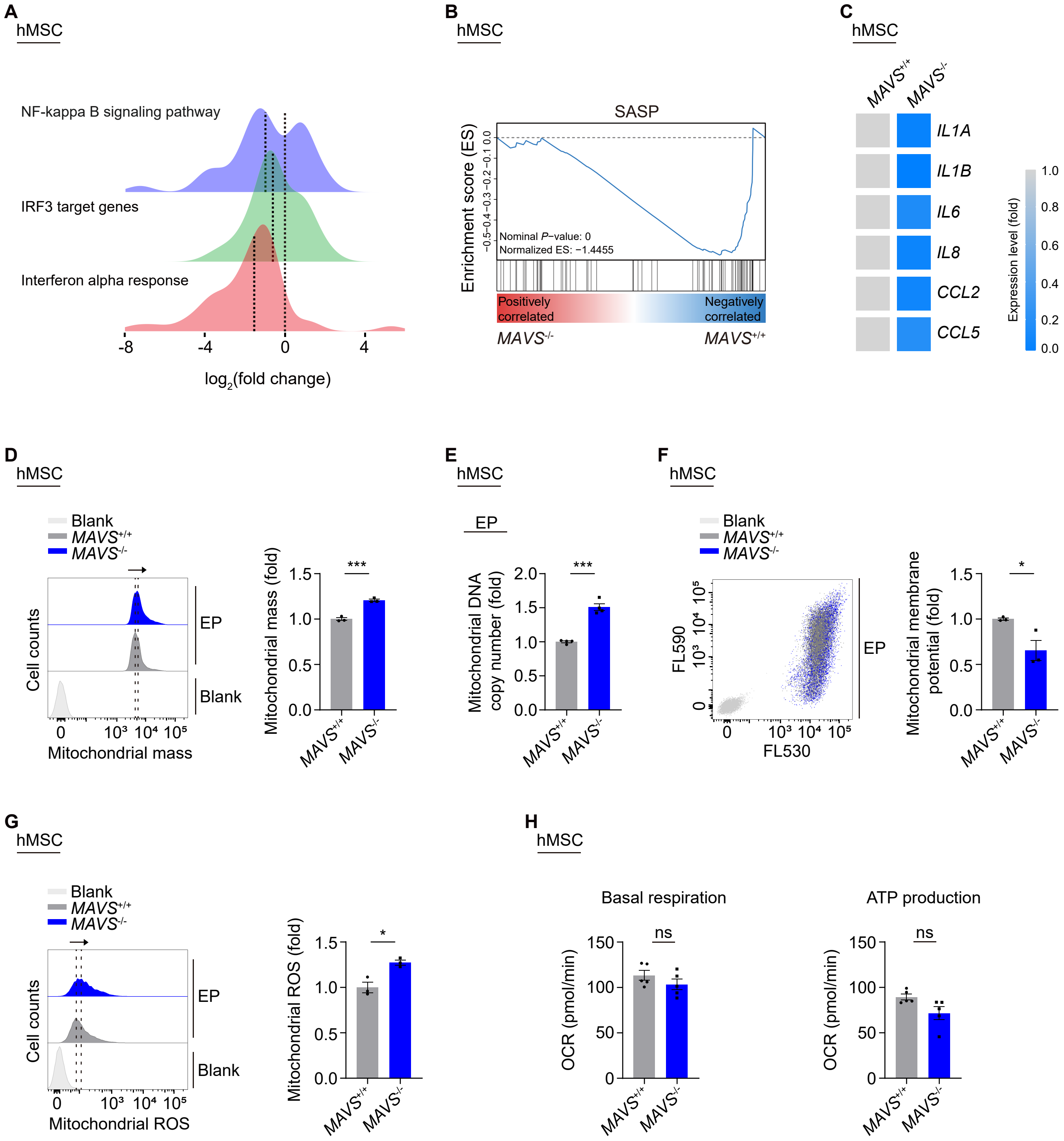
A
hMSC
NF-kappa B signaling pathway
IRF3 target genes
Interferon alpha response
-8
-4
0
4
log2(fold change)
B
hMSC
SASP
Enrichment score (ES)
Nominal P-value: 0
Normalized ES: −1.4455
Positively correlated
Negatively correlated
MAVS-/-
MAVS+/+
C
hMSC
MAVS+/+
MAVS-/-
IL1A
IL1B
IL6
IL8
CCL2
CCL5
Expression level (fold)
D
hMSC
Blank
MAVS+/+
MAVS-/-
Cell counts
EP
Blank
Mitochondrial mass
Mitochondrial mass (fold)
***
E
hMSC
EP
Mitochondrial DNA copy number (fold)
***
F
hMSC
FL590
FL530
Mitochondrial membrane potential (fold)
*
G
hMSC
Mitochondrial ROS
Mitochondrial ROS (fold)
H
hMSC
Basal respiration
ATP production
OCR (pmol/min)
ns

Figure S4

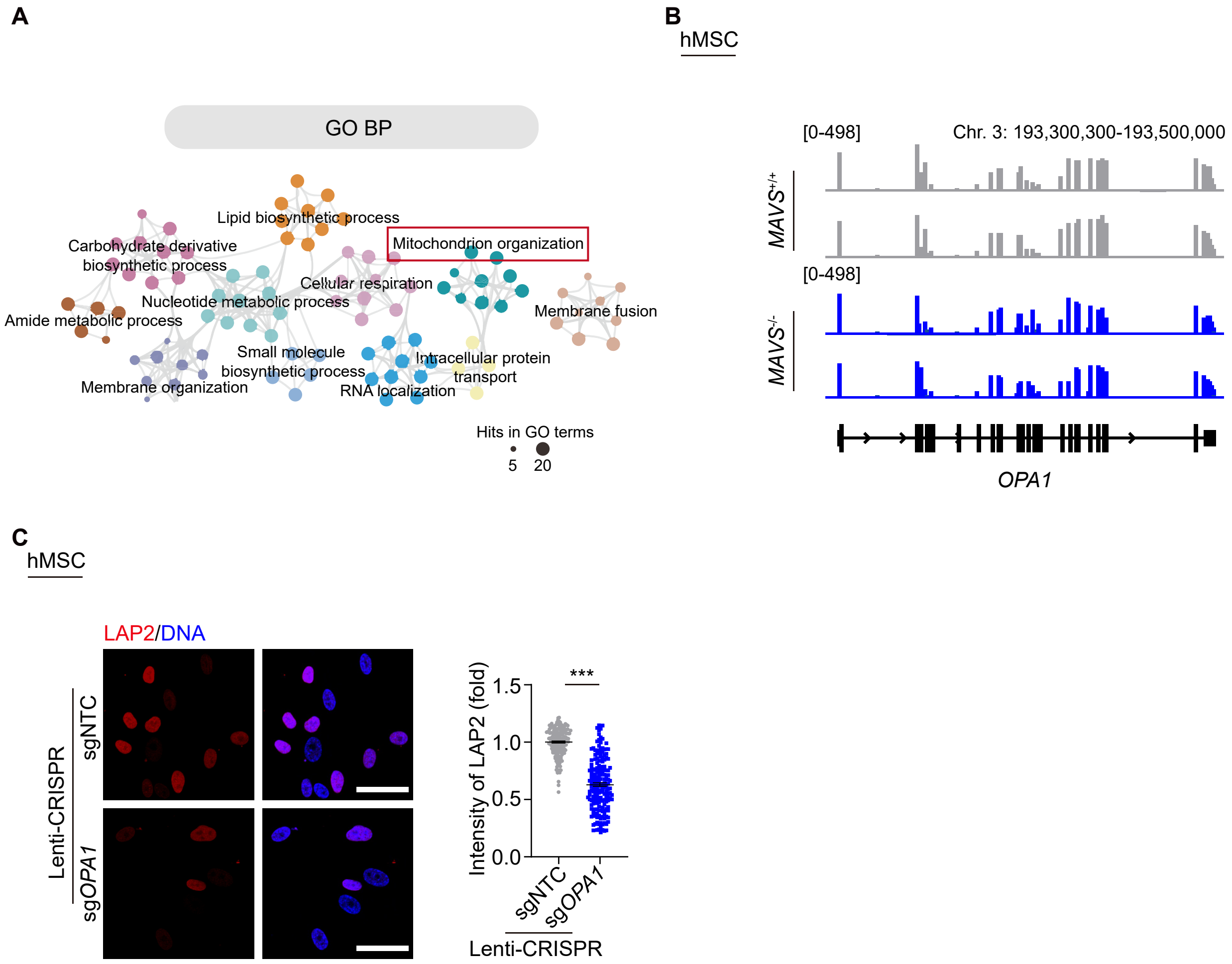
A
GO BP
Lipid biosynthetic process
Carbohydrate derivative
biosynthetic process
Mitochondrion organization
Cellular respiration
Nucleotide metabolic process
Amide metabolic process
Membrane fusion
Small molecule
biosynthetic process
Intracellular protein
transport
Membrane organization
RNA localization
Hits in GO terms
5
20
B
hMSC
[0-498]
Chr. 3: 193,300,300-193,500,000
MAVS+/+
[0-498]
MAVS-/-
OPA1
C
hMSC
LAP2/DNA
Lenti-CRISPR
sgNTC
sgOPA1
Intensity of LAP2 (fold)
1.5
1.0
0.5
0.0
***
sgNTC
sgOPA1
Lenti-CRISPR

Figure S5

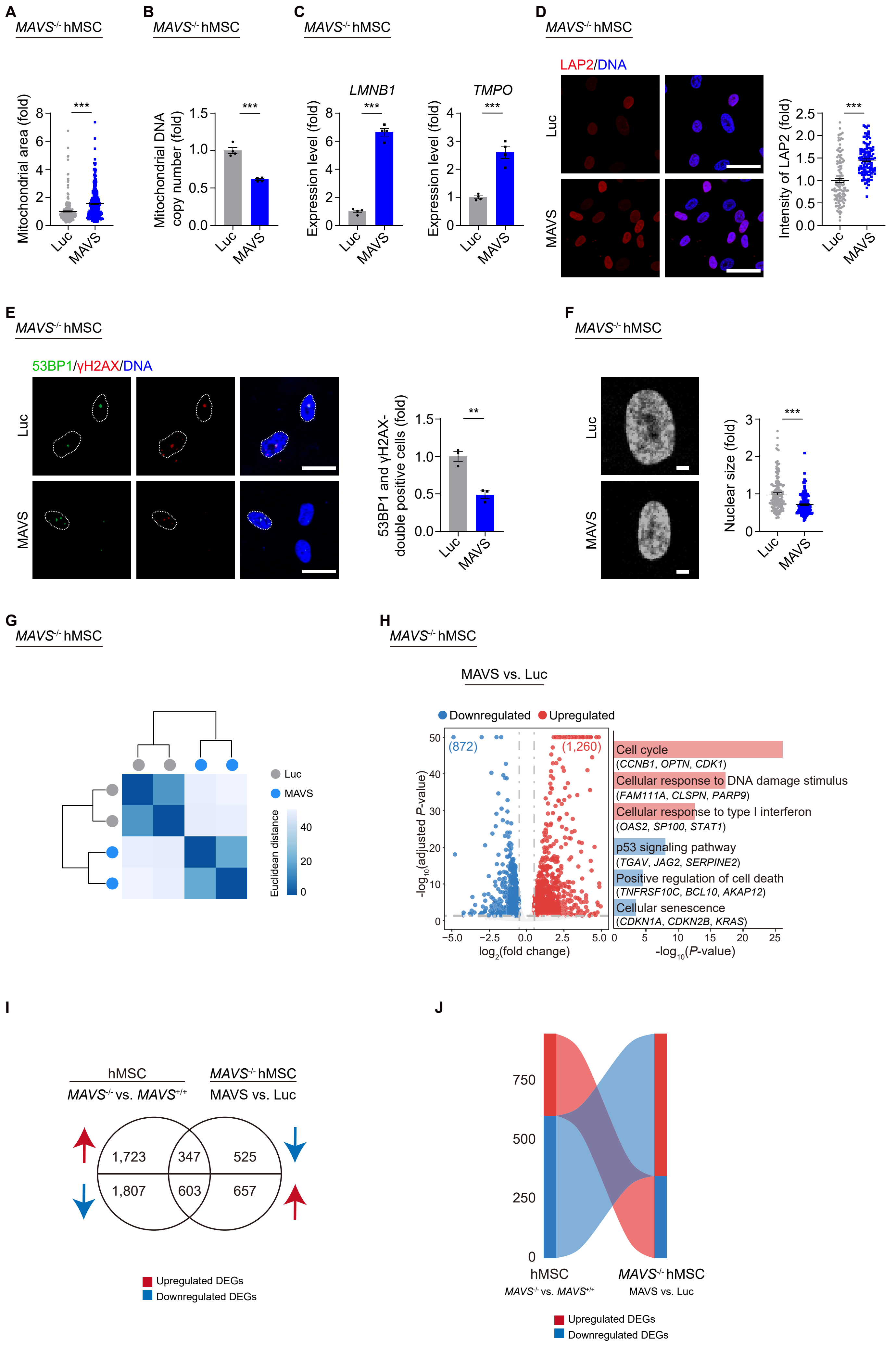

A
MAVS-/- hMSC
Mitochondrial area (fold)
***
Luc
MAVS
B
MAVS-/- hMSC
Mitochondrial DNA copy number (fold)
***
C
MAVS-/- hMSC
LMNB1
TMPO
Expression level (fold)
***
D
MAVS-/- hMSC
LAP2/DNA
Intensity of LAP2 (fold)
***
E
MAVS-/- hMSC
53BP1/γH2AX/DNA
53BP1 and γH2AX-double positive cells (fold)
**
F
MAVS-/- hMSC
Nuclear size (fold)
***
G
MAVS-/- hMSC
Luc
MAVS
Euclidean distance
H
MAVS-/- hMSC
MAVS vs. Luc
Downregulated
Upregulated
(872)
(1,260)
-log10(adjusted P-value)
log2(fold change)
Cell cycle
(CCNB1, OPTN, CDK1)
Cellular response to DNA damage stimulus
(FAM111A, CLSPN, PARP9)
Cellular response to type I interferon
(OAS2, SP100, STAT1)
p53 signaling pathway
(TGAV, JAG2, SERPINE2)
Positive regulation of cell death
(TNFRSF10C, BCL10, AKAP12)
Cellular senescence
(CDKN1A, CDKN2B, KRAS)
-log10(P-value)
I
hMSC
MAVS-/- vs. MAVS+/+
MAVS-/- hMSC
MAVS vs. Luc
1,723
347
525
1,807
603
657
Upregulated DEGs
Downregulated DEGs
J
hMSC
MAVS-/- vs. MAVS+/+
MAVS-/- hMSC
MAVS vs. Luc
Upregulated DEGs
Downregulated DEGs
